# Supplementary material for: Structural Analysis and Dynamic Processes of the Transmembrane Segment Inside Different Micellar Environments—Implications for the TM4 Fragment of the Bilitranslocase Protein
Source: Int J Mol Sci. 2019 Aug 26;20(17):4172. doi: 10.3390/ijms20174172 (PMC6747479; doi:10.3390/ijms20174172)
Supplement: Supplementary file 1 [file ijms-20-04172-s001.zip › ijms-560636-S/manuscript.pdf]

International Journal of
Molecular Sciences

MDPI

Article

Supplementary Materials: Structural Analysis and Dynamic Processes of the Transmembrane Segment Inside Different Micellar Environments—Implications for the TM4 Fragment of the Bilitranslocase Protein

Kosma Szutkowski [1], Emilia Sikorska [2], Iulia Bakanovych [3], Amrita Roy Choudhury [4,†], Andrej Perdih [4], Stefan Jurga [1], Marjana Novič [4,*] and Igor Zhukov [5,*]

1. NanoBioMedical Centre, Adam Mickiewicz University, Umultowska 85, 61-614 Poznań, Poland
2. Faculty of Chemistry, University of Gdańsk, Wita Stwosza 63, 80-308 Gdańsk, Poland
3. Institute of High Technologies, Taras Shevchenko National University, Volodymyrska 54, 01-601 Kyiv, Ukraine
4. National Institute of Chemistry, Hajdrihova 19, 1001 Ljubljana, Slovenia
5. Institute of Biochemistry and Biophysics, Polish Academy of Sciences, Pawińskiego 5a, 02-106 Warsaw, Poland

* Correspondence: marjana.novic@ki.si (M.N.); igor@ibb.waw.pl (I.Z.); Tel.: +48-22-592-2038 (I.Z.)

† Current address: Genialis d.o.o., Koprska 72, 1000 Ljubljana, Slovenia.

Received: date; Accepted: date; Published: date

check for updates

S1. Supporting Information

S1. Molecular dynamics simulation of the predicted BTL transmembrane α-helix TM4 and TM4A in the DPPC membrane for the initial stability assessment

Predicted BTL peptide TM4 / TM4A in lipid / water molecular systems were constructed using CHARMM [64] utilizing the methodology presented in our previous studies of the TM2 and TM3 segments of BTL [15,16]. The amino acid sequence that was predicted to encompass the BTL transmembrane region of the TM4 helix was the sequence of 20 BTL residues: 258–277 [14] followed by the prediction model with statistics sequence comprising 23 BTL residues: 254–276. Two three dimensional models were generated by the available CHARMM topology and structural libraries [65,66]. The α-helix conformation for each sequence was generated by constraining the backbone torsion angles ϕ and ψ to the values of -57° and -47° for each amino acid backbone torsion angle [87]. Analogously, [15,16] the additional amino acids corresponding to the residues located prior to the start (256–257 residues for TM4 system and 252–253 residues from the TM4A system) and subsequent to the end of the transmembrane α-helix (278–279 residues for TM4 system and 277–278 residues from the TM4A system) on the BTL sequences were added on the C-terminal and N-terminal end of TM4 and TM4A. These additional amino acid residues were not constrained to the α-helical conformation.

S2. Circular Dichroism spectroscopy of TM4 transmembrane fragment in DPC and SDS micellar environments

Circular Dichroism secondary structure analysis was performed at 298 K on 0.15 mg/mL peptide solutions using Jasco J-815 spectropolarimeter. The pH of the measured solutions was between 6.5, and 7.0 checked just before starting the experiments. Every spectrum was averaged from three independent runs. CD spectra were acquired in the surfactant-free aqueous solution, trifluoroethanol (TFE), micellar aqueous solutions of SDS and DPC, and micellar solution of DPC in H_2O/CH_3OH (v:v ∼ 7:3). Finally, the data were corrected by subtracting the background and analyzed as mean residue molar ellipticity

Θ (degree $\times$ cm^2 $\times$ dmol^{-1}) vs. wavelength λ (nm). The content of the secondary structure was calculated from the spectra using a CONTINLL method [88] using as a reference the database of 37 soluble and 13 membrane proteins (SMP50) with precisely known secondary structures.

Measurement of the ^{15}N relaxation data for ^{15}N-labeled Ala261 in the TM4 fragment in SDS-d_{25} and DPC-d_{38} micelle

Incorporation of ^{15}N-labeled residues in studies peptide dramatically expand our knowledge about molecular dynamic processes in the backbone. the ^{15}N relaxation data were acquired for ^{15}N-labeled Ala261 in two available magnetic fields (14.1 T and 18.8 T) at 303 K for TM4 fragment in SDS-d_{25} and DPC-d_{38} environments. The experimental data were recorded using pulse sequence presented in BioPack (Agilent Inc. PaloAlto CA, USA) written on base previously published studies. [85] The ^{15}N R_1 relaxation rates on 18.8 T were extracted from experiment conducted as eight delays – 10, 90, 170. 290, 410, 550, 690 and 850 ms – in SDS-d_{25} and DPC-d_{38} surfactants. The similar experiments on 14.1 T were performed as a nine points – 10, 90, 170. 290, 410, 550, 690, 850 and 1010 ms – for TM4 in SDS-d_{25}, and as a ten points – 10, 90, 170. 290, 410, 550, 690, 850, 1010 and 1025 ms – for TM4 in DPC-d_{38} media. The ^{15}N R_2 relaxation rates on 18.8 T were acquired with eight delay – 10, 30, 50, 70, 90, 130, 170 and 210 ms – for ^{15}N-labeled Ala261 in SDS-d_{25} and DPC-d_{38} micelle. Due to fast relaxation for TM4 in DPC-d_{38} the values of R_2 relaxation rate was obtained only on base five points (Figure 5B in the main text). The data concerned steady-state ^{1}H – ^{15}N *NOE* on 14.1 T and 18.8 T were not possible to obtain, probably due to low concentration of TM4 peptide for that experiments.

S3. Carver-Richards model for two exchangeable sites from ^{31}P relaxation measurements

To explore the slow dynamic processes in DPC surfactant, the ^{31}P R_2 CPMG experiment. The obtained data were analyzed with Carver-Richards model [52] described two—site conformational exchange processes [51]:

$$T_2^{-1} = -\frac{1}{\tau_{cp}} \ln \lambda \tag{S1}$$

where

$$\ln \lambda = -\tau_{cp}\frac{\alpha_+}{2} + \ln\left\{(D_+ \cosh^2 \xi - D_- \cos^2 \eta)^{\frac{1}{2}} + (D_+ \sinh^2 \xi + D_- \sin^2 \eta)^{\frac{1}{2}}\right\}$$

$$2D_{\pm} = 1 \pm \frac{(\Psi + 2\Delta\omega^2)}{(\Psi^2 + \zeta^2)^{\frac{1}{2}}}$$

$$\xi = \frac{\tau_{cp}}{2\sqrt{2}}\left\{\pm\left[+\Psi + (\Psi^2 + \zeta^2)^{\frac{1}{2}}\right]^{\frac{1}{2}}\right\}$$

$$\eta = \frac{\tau_{cp}}{2\sqrt{2}}\left\{\pm\left[-\Psi + (\Psi^2 + \zeta^2)^{\frac{1}{2}}\right]^{\frac{1}{2}}\right\}$$

$$\Psi = \alpha_-^2 - \Delta\omega^2 + \frac{4}{\tau_a\tau_b}$$

$$\zeta = 2\Delta\omega^2\alpha_-$$

$$\alpha_- = \frac{1}{T_{2a}} - \frac{1}{T_{2b}} + \frac{1}{\tau_a} - \frac{1}{\tau_b}$$

$$\alpha_+ = \frac{1}{T_{2a}} + \frac{1}{T_{2b}} + \frac{1}{\tau_a} + \frac{1}{\tau_b}$$

$$\Delta\omega = \omega_a - \omega_b$$

$$\omega_{(a,b)} = 2\pi\delta_{(a,b)}\omega_0$$

Life times in site $< A >$, τ_a and site $< B >$, τ_b, populations P_a and P_b and k_m obey to the following relations

$$P_a + P_b = 1$$
$$\frac{P_a}{\tau_a} = \frac{P_b}{\tau_b} = \frac{1}{\tau} = k_m$$

Table S1. The comparison of predictions of transmembrane regions of bilitranslocase using PredαTM algorithm [22] with other algorithms.

Algorithm	Number of predicted transmembrane regions	Predicted transmembrane regions
PredαTM	4 (TM regions 1, 2, 3, 4)	24 – 48, 75 – 94, 220 – 238, 254 – 276
TMpred	4 (TM regions 1, 2, 3, 4)	26 – 45, 75 – 102, 217 – 237, 256 – 278
TopPred II	4 (TM regions 1, 2, 3, 4)	26 – 46, 72 – 92, 221 – 241, 257 – 277
SOUSI	0	
PRED-TMR	3 (TM regions 1, 2, 4)	27 – 46, 75 – 94, 256 – 277
TMHMM	2 (TM regions 1, 4)	20 – 42, 256 – 278
HMMTOP	3 (TM regions 1, 3, 4)	20 – 43, 226 – 245, 257 – 277
Phobius	2 (TM regions 1, 4)	20 – 41, 256 – 277
SVMtm	2 (TM regions 1, 4)	27 – 41, 257 – 273
DAS-TMfilter	2 (TM regions 1, 4)	27 – 42, 257 – 271
MEMSAT	2 (TM regions 1, 4)	22 – 42, 257 – 275
SCAMPI	3 (TM regions 1, 3, 4)	21 – 41, 221 – 241, 256 – 276
MemBrain	3 (TM regions 1, 2, 4)	23 – 42, 74 – 82, 256 – 270
Philius	3 (TM regions 1, 2, 4)	19 – 41, 76 – 99, 255 – 279
OCTOPUS	2 (TM regions 1, 4)	23 – 43, 254 – 274
TOPCONS	3 (TM regions 1, 3, 4)	21 – 41, 221 – 241, 259 – 279

Table S2. The values of force constants (kcal/mol/Å^2) used during the equilibration of the TM4 and TM4A helices in the DPPC membrane [15,16,89].

Equilibration						
Stage	step 1	step 2	step 3	step 4	step 5	step 6
K_1	10	5.0	2.5	1.0	0.5	0.1
K_2	5.0	2.5	1.0	0.5	0.1	0.0
K_{wforce}	2.5	2.5	1.0	0.5	0.1	0.0
K_{tforce}	2.5	2.5	1.0	0.5	0.1	0.0
K_{mforce}	2.5	2.5	1.0	0.5	0.1	0.0

K_1 - the force constant applied to the protein backbone
K_2 - the force constant applied to the side chains
K_{wforce} - the force constant applied to keep water molecules away from the hydrophobic core
K_{tforce} - the force constant applied to the lipid tail
K_{mforce} - the force constant applied to the movement of the lipid head

Table S3. Structural statistics of distance constraints used for evaluation of the TM4 fragment in SDS and DPC micelle. NMR restraints and structural statistics for the ensemble of 20 lowest-energy structures of TM4 fragment in SDS-d_{25} and DPC-d_{38} surfactants.

	SDS-d_{25}	DPC-d_{38}
NOE distance constraints	258	278
Intra-residue $\|i\text{-}j\|= 0$	124	170
Sequential $\|i\text{-}j\|= 1$	113	88
Medium range $\|i\text{-}j\|\leq 5$	21	20
Hydrogen bonds		
Distance constraints	12	16
Torsion angles restraints		
Backbone (ϕ/ψ)	8	12
Ramachandran plot		
Residues in most-favored regions (%)	82.5	85.0
Residues in additional allowed regions (%)	17.2	14.8
Residues in generously allowed regions (%)	0.2	0.2
Residues in disallowed regions (%)	0.0	0.0
RMSD to the mean co-ordinates		
Ordered backbone atoms ($\dot{A}$)	0.23 ± 0.06[a]	0.18 ± 0.05[b]
Ordered side-chains atoms ($\dot{A}$)	0.91 ± 0.16	0.94 ± 0.17
RMS Z-scores[c]		
Bond lengths ($\dot{A}$)	0.253 ± 0.185	0.127 ± 0.310
Bond angles (^{o})	0.950 ± 0.143	0.966 ± 0.129
Side chain planarity	1.252 ± 0.049	1.228 ± 0.050
Nonbonded of VdW and Coulomb energies	-1.144 ± 0.158	0.429 ± 0.108
Structure Z-scores[c]		
First-generation packing quality	-1.110 ± 0.437	0.333 ± 0.355
Second-generation packing quality	-1.651 ± 0.517	-0.353 ± 0.374

[a] region Pro258 — Leu266
[b] region Pro258 — Met268
[c] evaluated with WhatIf software [90]

Table S4. Diffusion measurements were performed on ^{1}H, ^{2}H and ^{31}P isotopes at 303 K.

Micellar media	^{1}H D_{tr} 10^{-11} (m^2/s)[a]	R_h (Å)[b]	^{2}H D_{tr} 10^{-11} (m^2/s)[c]	R_h (Å)	^{31}P D_{tr} 10^{-11} (m^2/s)[c]	R_h (Å)
SDS-d_{25}			7.54 ± 0.01	37		
SDS-d_{25} + TM4	8.24 ± 0.06	34				
DPC-d_{38}			7.30 ± 0.20	38	6.97 ± 0.02	40
DPC-d_{38} + TM4	8.19 ± 0.07	34			6.98 ± 0.22	40

[a]Experimental data obtained on 18.8 T
[b]Hydrodynamic radius calculated with Stokes-Einstein equation
[c]Experimental data recorded on 14 T

Table S5. ^{31}P relaxation data obtained on 14.1 T magnetic field at 303K with and without TM4 segment inside DPC-d_{38} micelle.

Media	R_1 (s^{-1})	R_2 (s^{-1})
DPC-d_{38}	0.933 ± 0.003	7.874 ± 0.062
DPC-d_{38} + TM4	0.959 ± 0.005	83.333 ± 13.889

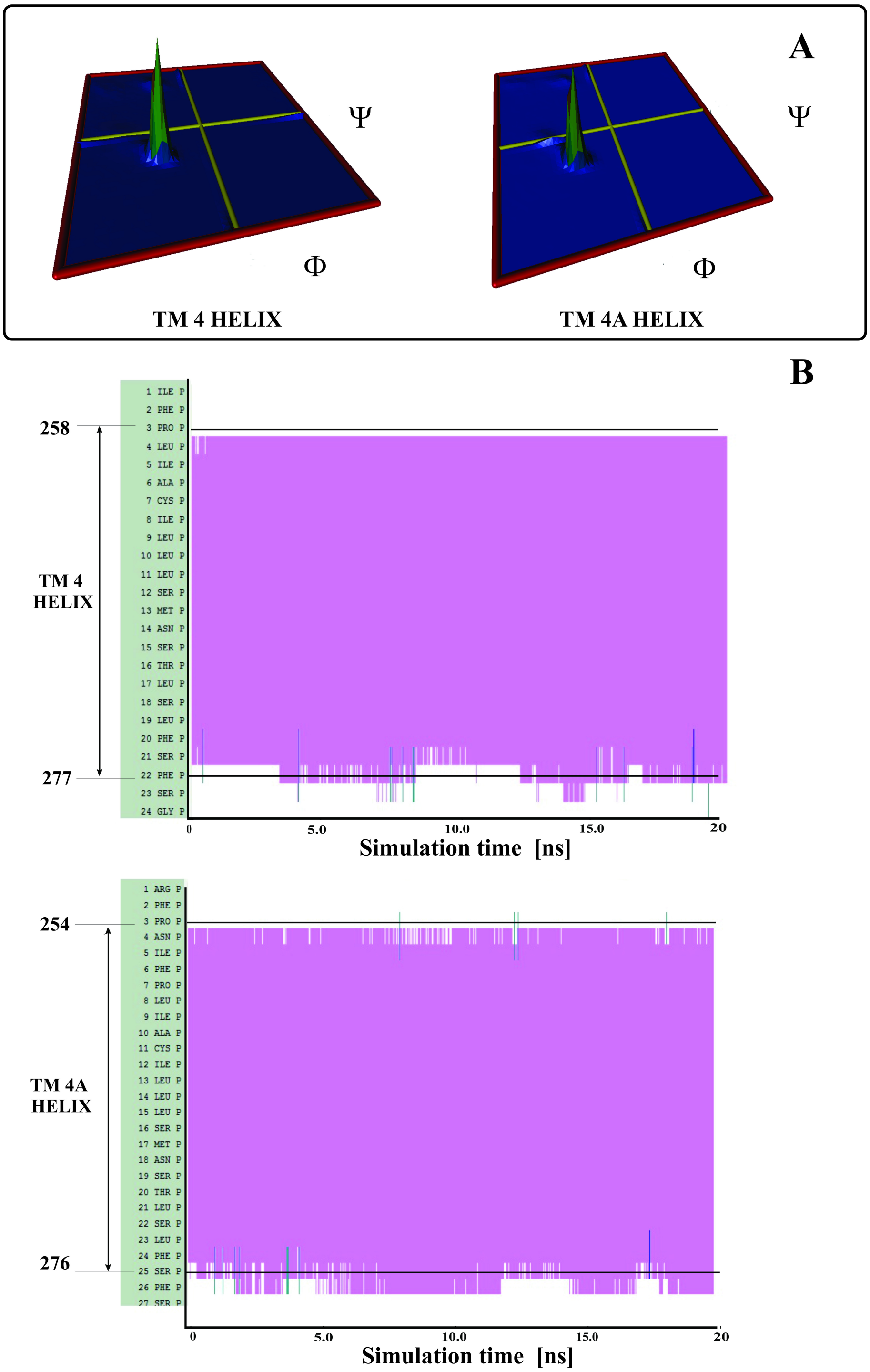

Figure S1. The 3D Ramachandran diagrams for the backbone torsion angles and secondary structural analysis. (**A**) The backbone torsion angles ϕ and ψ of the predicted residues of the transmembrane helix (generated for residues 258–277 TM4 helix and 254–277 TM4A helices. (**B**)) Two-dimensional time plots of the secondary structure analysis. Purple color depicts the presence of the α-helical structure, rarely occurring green lines indicate the presence of turns while the blue lines indicate the presence of the 3_{10}-helical structures (only a few frames). Residue numbers from 3–22 or 3–25 on the y-axis correspond to the 258–277 or 254–276 residues of the BTL TM4 or TM4A sequences respectively.

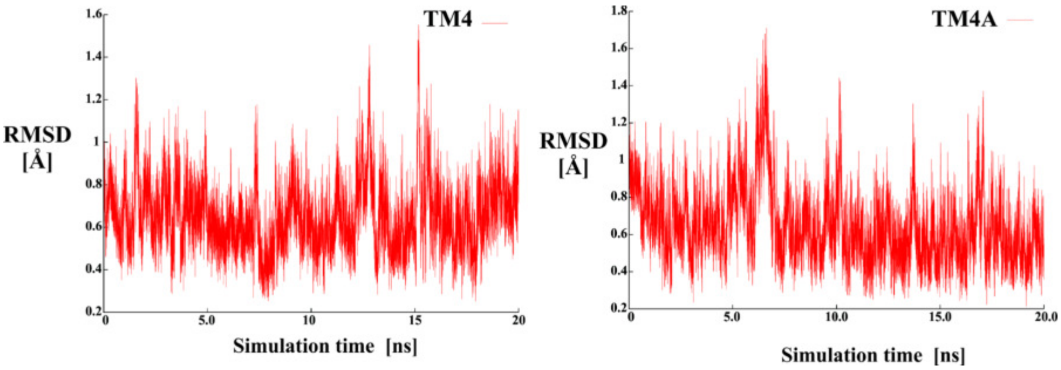

Figure S2. RMSD during MD simulations. RMSD time graphs of the backbone atoms for the α-helices TM4 (initial prediction) and TM4A (prediction with statistics).

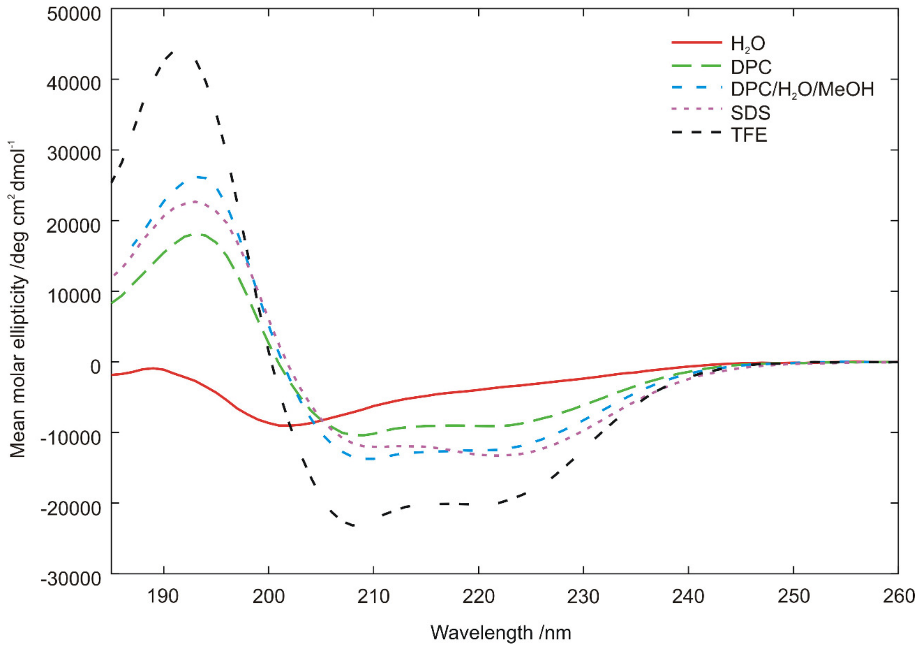

Figure S3. CD spectra of the TM4 fragment. CD spectra of TM4 fragment recorded at 298 K in different media: aqueous solution (red), TFE (black), SDS (pink), DPC (green), and DPC in H_2O/CH_3OH (blue).

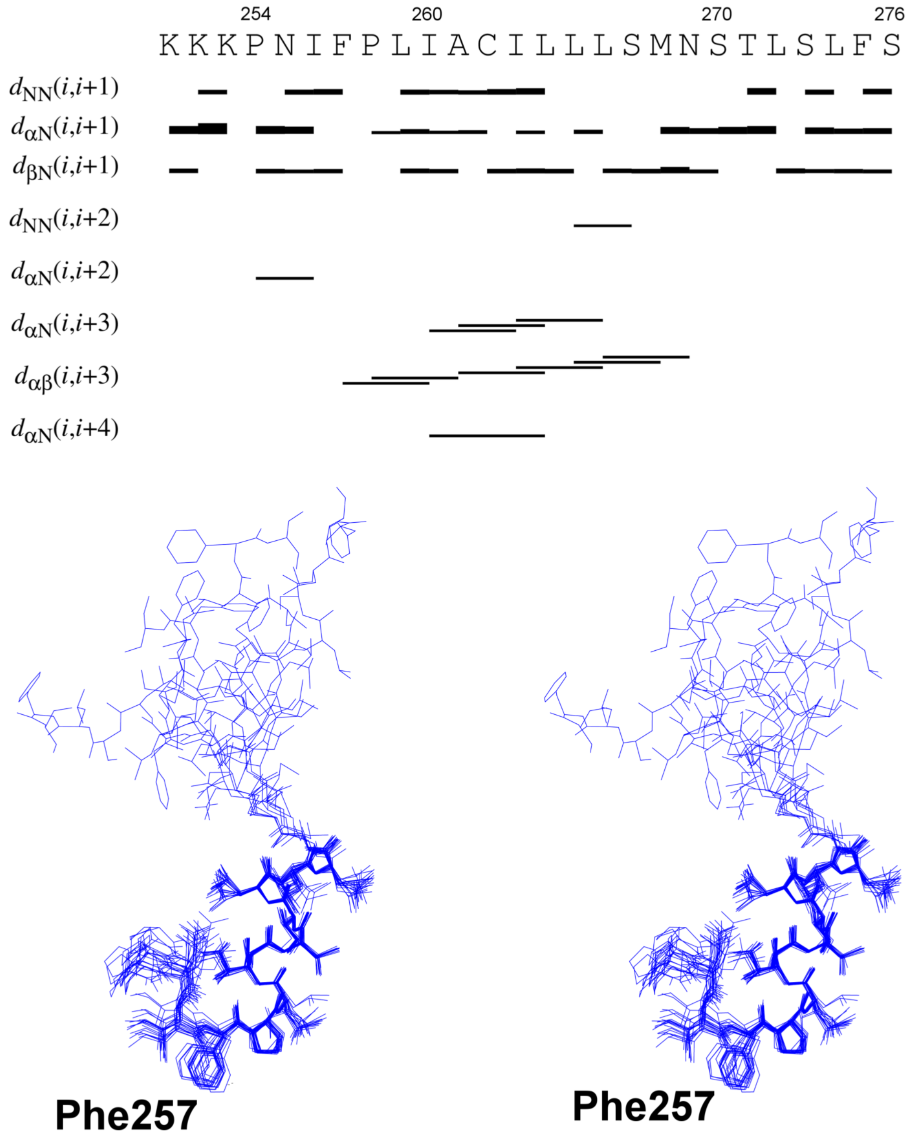

Figure S4. The backbone distance constraints and stereo view of TM4 in SDS-d_{25} micelle. The sequence plot of a backbone $^{1}H - ^{1}H$ distance constraints yielded from the analysis of 2D NOESY experiment in SDS-d_{25} micelle (**up**). Stereo view of the high-resolution 3D structure of TM4 evaluated with CYANA software (**down**).

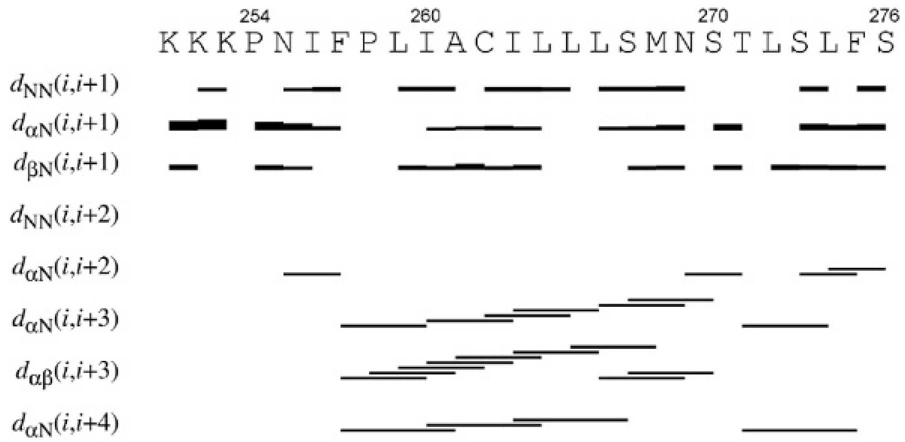

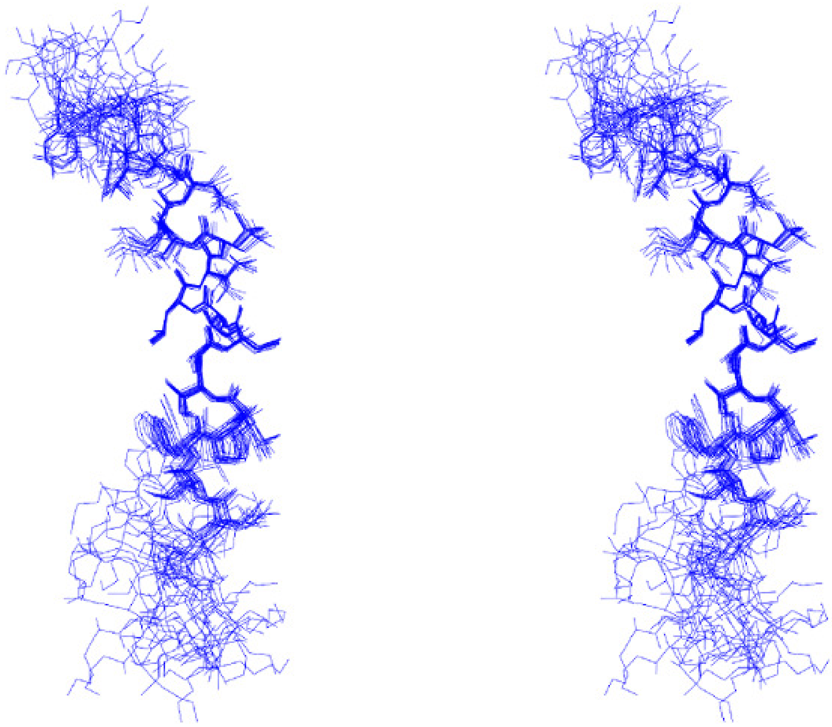

Figure S5. The backbone distance constraints and stereo view of TM4 in DPC-d_{38} micelle. Sequence plot of backbone ^1H – ^1H distance constraints yielded from analysis of 2D NOESY experiment in DPC-d_{38} micelle (**up**). Stereo view of the high-resolution 3D structure of TM4 evaluated with CYANA software (**down**).

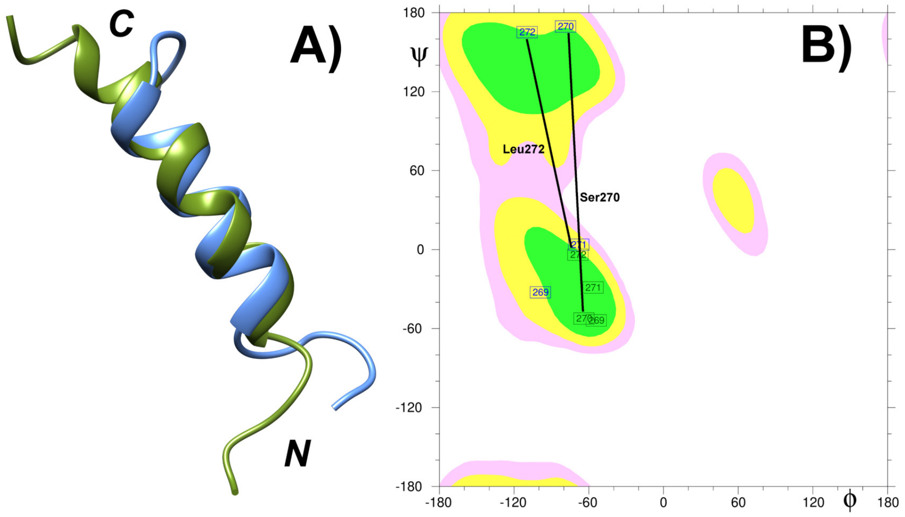

Figure S6. (**A**) Comparison of the 3D structures of the TM4 peptide in SDS (blue) and DPC (green) obtained on the basis of NMR data. The orientation of α-helices are shown by N- and C-termini. (**B**) The N-terminal (Phe257) and C-terminal (Ser270, Thr271 and Leu272) residues, demonstrated structural alterations in anionic (SDS) and zwitterionic (DPC) media. Changes for ϕ and ψ torsion angles are highlighted.

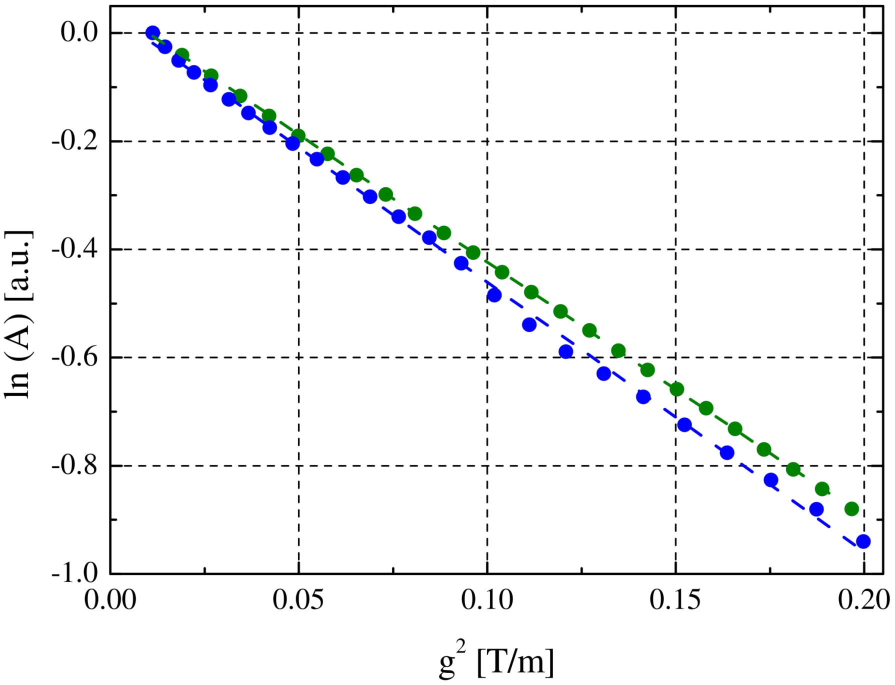

Figure S7. The ^{1}H PGSE spin echo attenuation obtained for the TM4 peptide in SDS-d_{25} (blue) and DPC-d_{38} (green) micelles at 303 K. The obtained value of D_{tr} coefficient for TM4 fragment in SDS-d_{25} surfactant is similar to reported previously by our group [17].

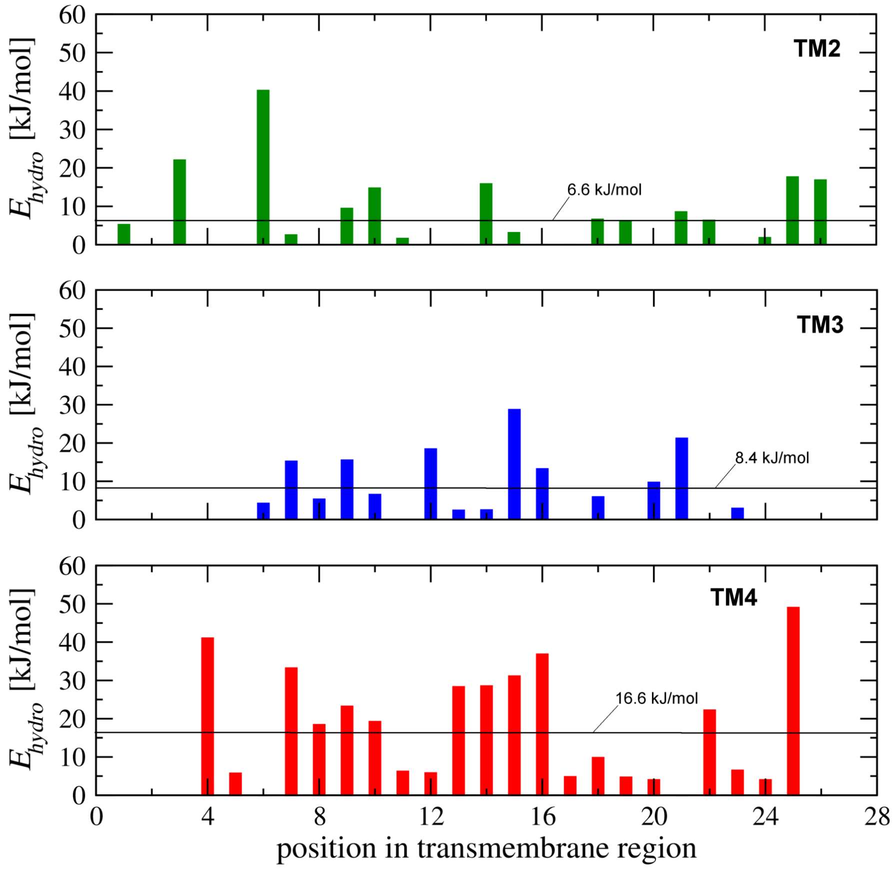

Figure S8. Energy of hydrophobic interactions of the TM2, TM3 and TM4 transmembrane fragments with SDS micelle. Energy of hydrophobic interactions of the transmembrane domains of BTL protein with SDS-d_{25} micelle in residue specific manner. The data were calculated using previously evaluated 3D structures TM2 [16], TM3 [15], and TM4 (this work) peptides with YASARA software using AMBER14 force field. Presented alignment of the TM3 peptide has reverse order due to orientation in membrane in respect to the TM2 and TM4 fragments.

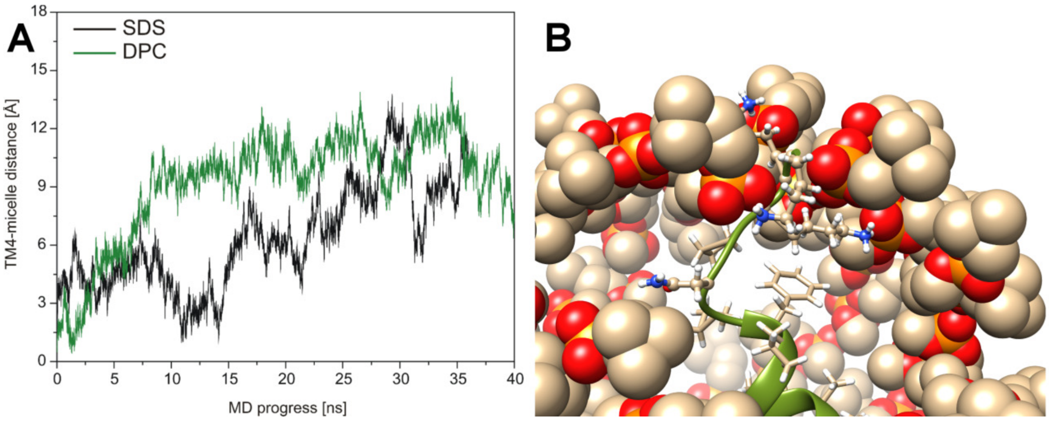

Figure S9. The time dependence of the distance between TM4 peptide and micelle center of mass. (**A**) The time dependence of the distance between a center of mass of TM4 peptide in SDS and DPC micelle during MD simulations. In both cases TM4 segment 'flows' from the center towards to the surface of a micelles. (**B**) Effect of snorkeling for N-terminal lysines with phosphate groups located on surface of DPC micelle. The phosphocholine part of DPC monomers are presented as balls and side chains of residues from TM4 fragment shown as balls and sticks.

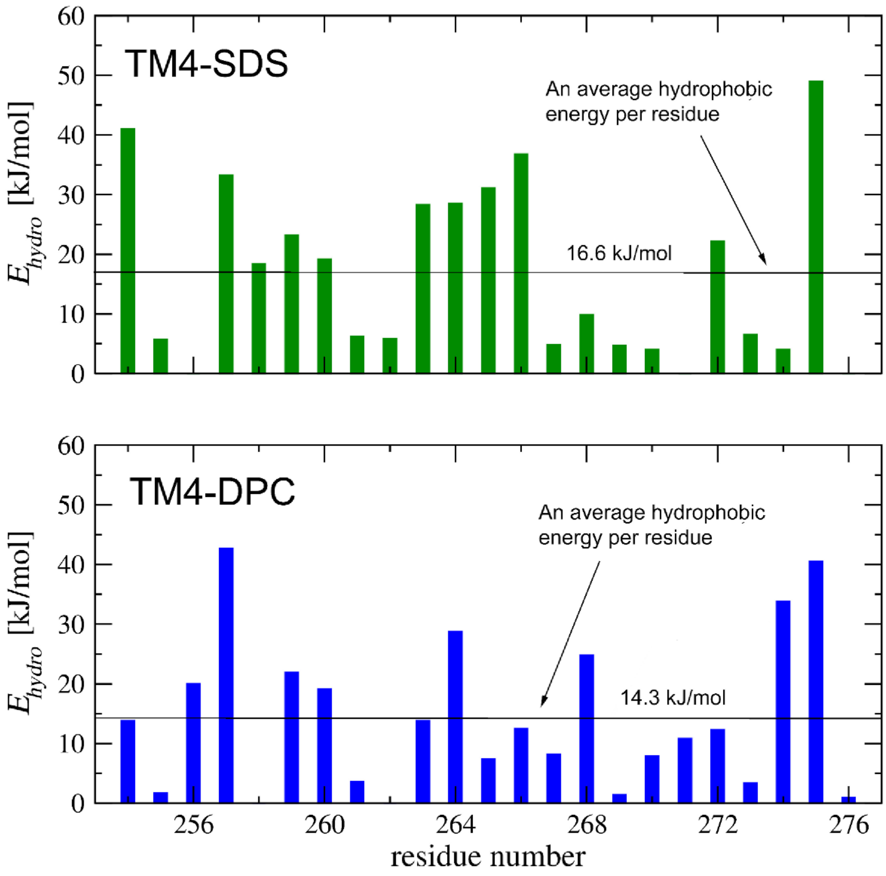

Figure S10. Energy of hydrophobic interactions of the TM4 transmembrane fragment with SDS and DPC micelle. The energy of hydrophobic interactions (ΔH_w^m) spanning over all residues of the TM4 segment in the SDS-d_{25} and DPC-d_{38} micelles obtained from the NMR solved 3D structures in Figure 7. The energies were calculated with YASARA software and AMBER 2014 force field. An average energy (per residue) is depicted as the horizontal line.

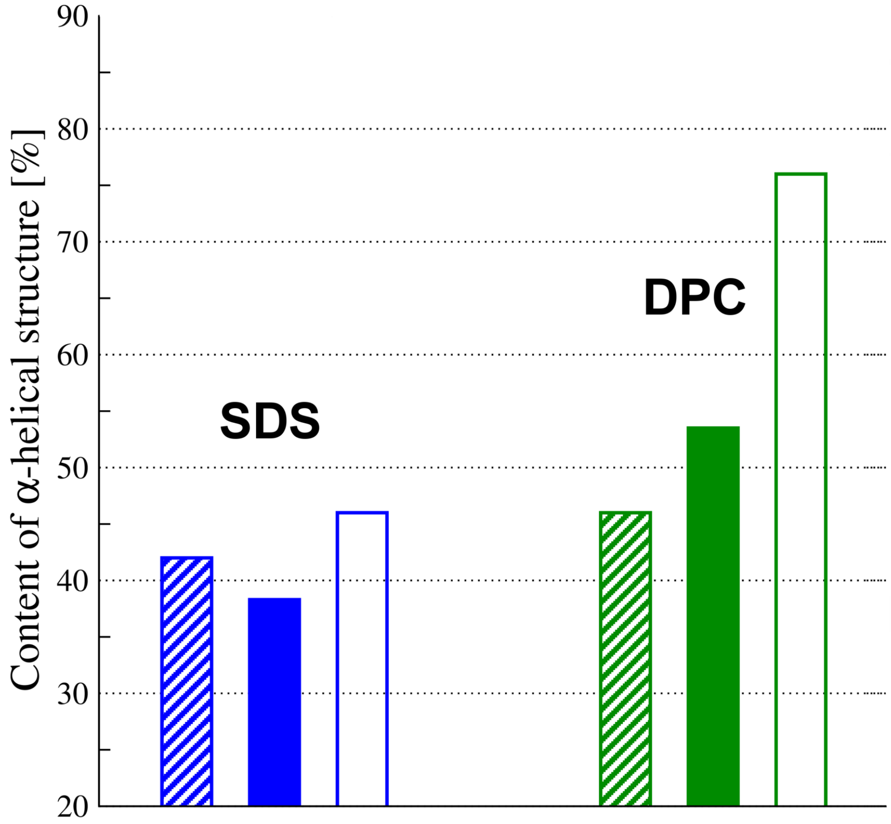

Figure S11. Content of the α-helical conformation in the TM4 fragment defined with CD measurements (shadow), NMR (filled) and molecular dynamic simulations (empty) procedures in SDS (blue) and DPC (green) surfactants.

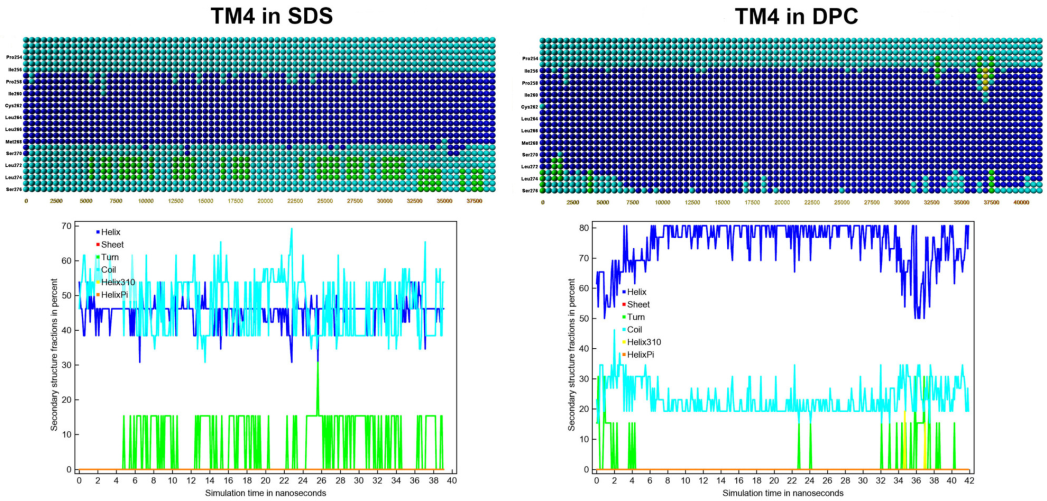

Figure S12. Time plot of the of the secondary structure analysis for the TM4 fragment in SDS-d_{25} and DPC-d_{38} micelle obtained during molecular dynamic simulations performed in Yasara software.

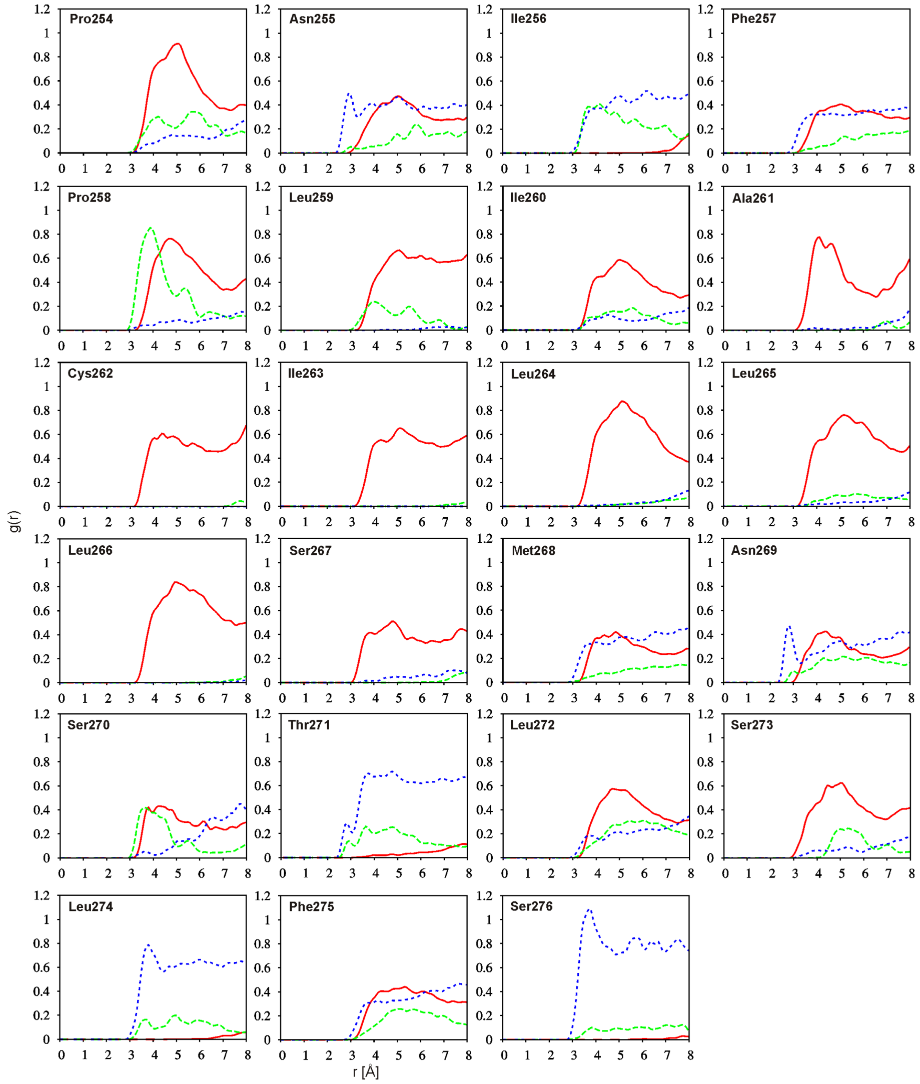

Figure S13. Radial distribution functions (RDF) plots. RDF obtained with *Ptraj* program for TM4 peptide in SDS-d_{25} micelle. RDF of hydrophobic (red), hydrophilic (green) and water molecules (blue) calculated for heavy atoms in the side chains.

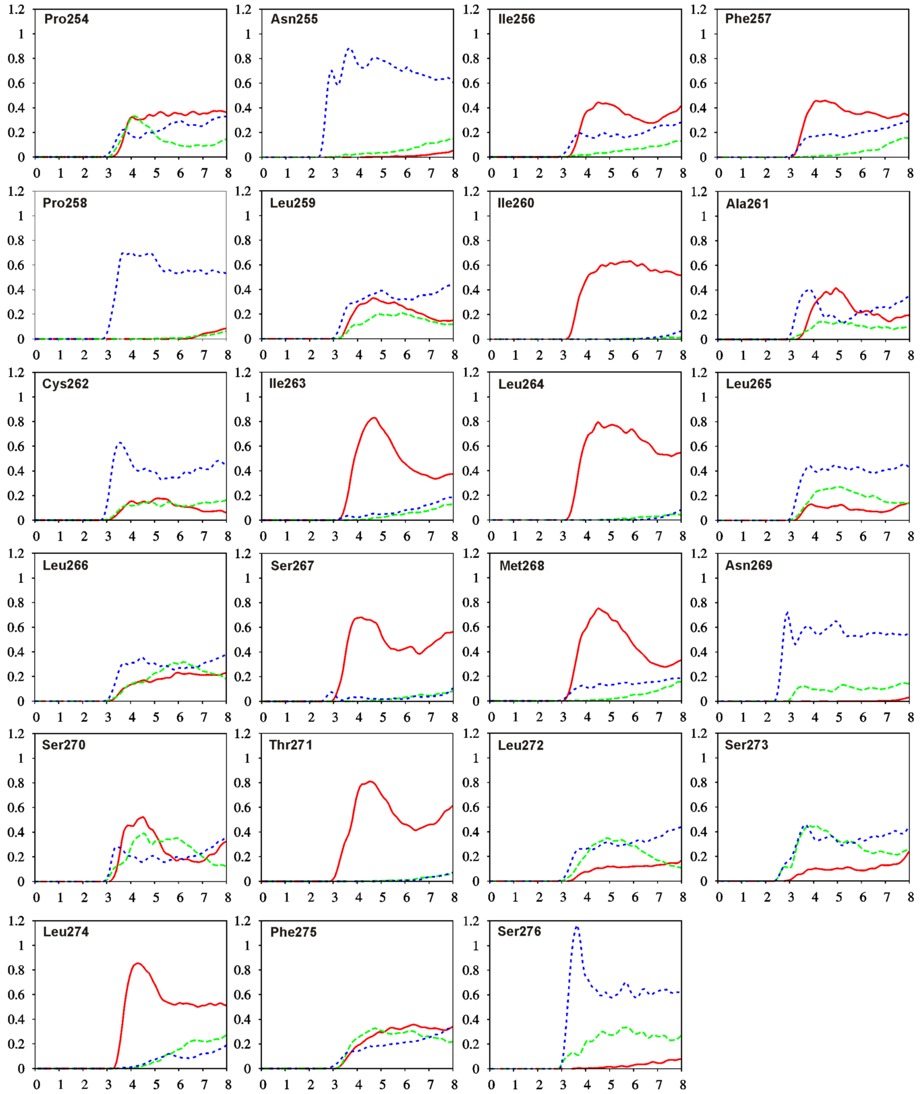

Figure S14. Radial distribution functions (RDF) plots. RDF plots obtained with *Ptraj* program for TM4 peptide in DPC-d_{38} micelle. RDF of hydrophobic (red), hydrophilic (green) and water molecules (blue) calculated for heavy atoms in the side chains.

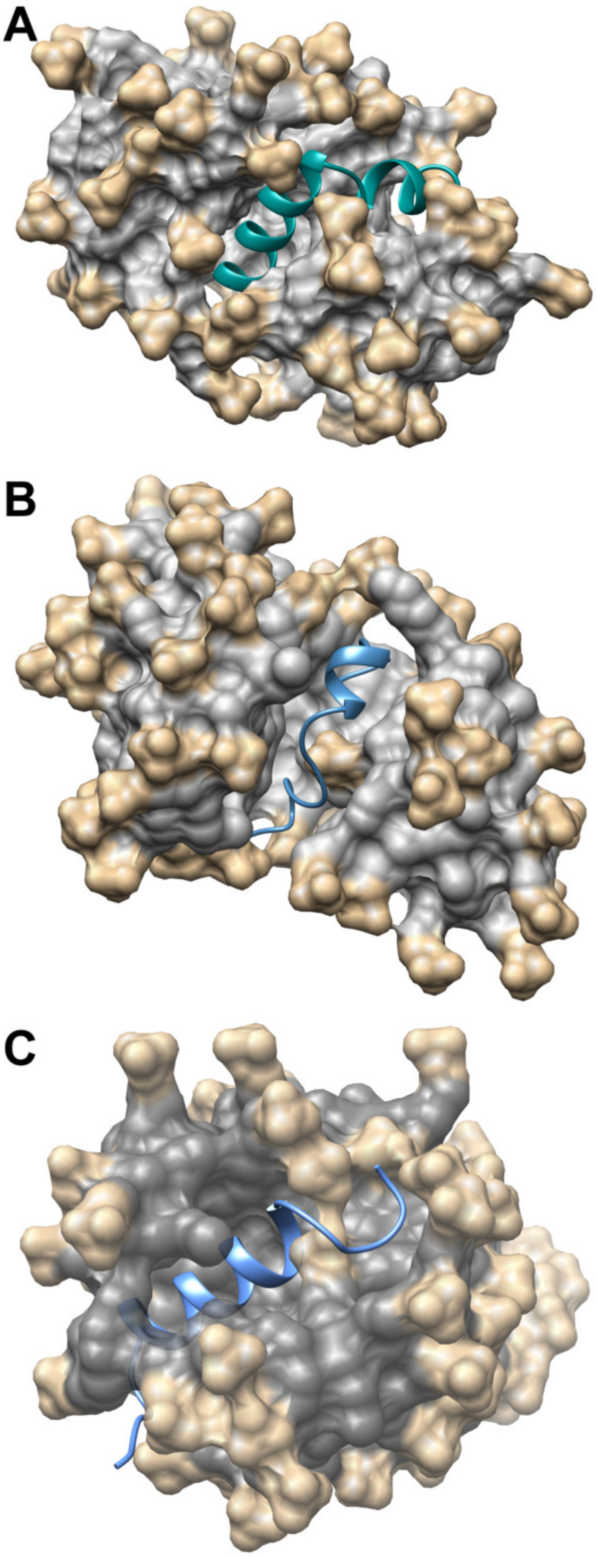

Figure S15. The 3D structure of (**A**) TM2 [16], (**B**) TM3 [15] and (**C**) TM4 transmembrane fragments of BTL protein in SDS-d_{25} micelar media solved with NMR spectroscopy.

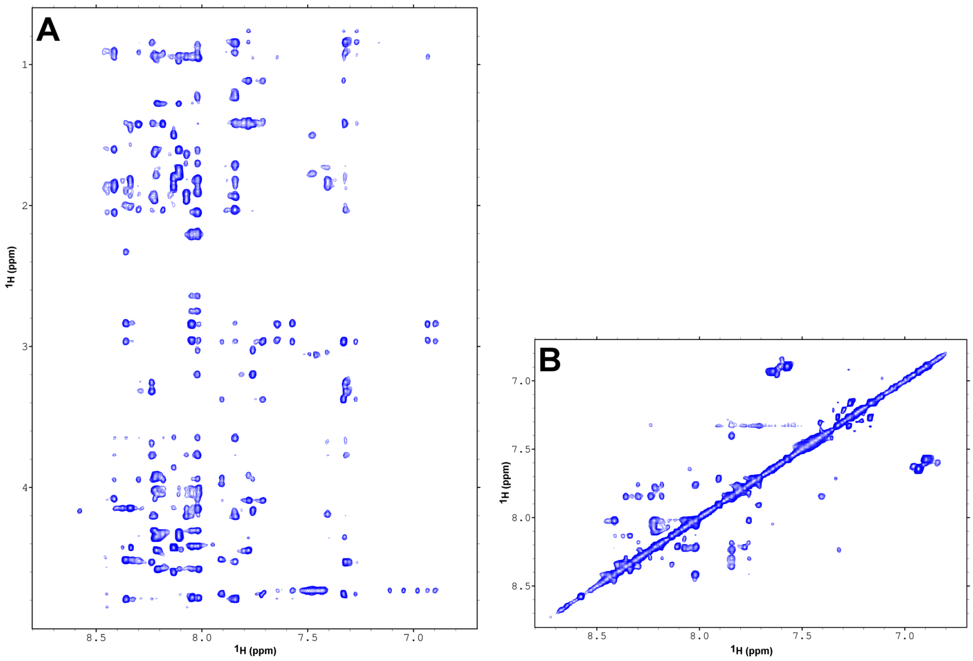

Figure S16. The fragments of 2D ^{1}H—^{1}H NOESY spectrum acquired for TM4 fragment in SDS-d_{25} micelle. The 2D homonuclear ^{1}H—^{1}H NOESY data collected with mixing time 120 ms for TM4 fragment in SDS-d_{25} micelle at 303 K on Agilent DDR2 800 NMR spectrometer. (**A**) – correlations between amide and aliphatic protons are shown; (**B**) – the region characteristic for amide protons.

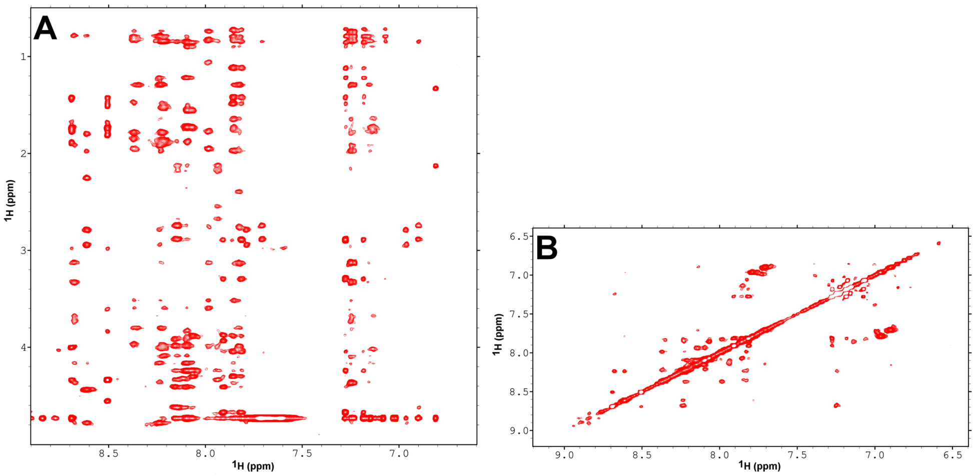

Figure S17. The fragments of 2D ^{1}H–^{1}H NOESY spectrum acquired for TM4 fragment in DPC-d_{38} micelle. The 2D homonuclear ^{1}H—^{1}H NOESY data collected with mixing time 120 ms for TM4 fragment in DPC-d_{38} micelle at 303 K on Agilent DDR2 800 NMR spectrometer. (**A**) – correlations between amide and aliphatic protons are shown; (**B**) – the region characteristic for amide protons.

References

1. Tzakos, A.G.; Briasoulis, E.; Thalhammer, T.; Jäger, W.; Apostolopoulos, V. Novel oncology therapeutics: Targeted drug delivery for cancer. *J. Drug Deliv.* **2013**, *2013*, 918304.
2. Buxhofer-Ausch, V.; Secky, L.; Wlcek, K.; Svoboda, M.; Kounnis, V.; Briasoulis, E.; Tzakos, A.G.; Jaeger, W.; Thalhammer, T. Tumor-specific expression of organic anion-transporting polypeptides: transporters as novel targets for cancer therapy. *J. Drug Deliv.* **2013**, *2013*, 863539.
3. Passamonti, S.; Terdoslavich, M.; Franca, R.; Vanzo, A.; Tramer, F.; Braidot, E.; Petrussa, E.; Vianello, A. Bioavailability of flavonoids: A review of their membrane transport and the function of bilitranslocase in animal and plant organisms. *Curr. Drug Metab.* **2009**, *10*, 369–394.
4. Terdoslavich, M.; de Graaf, I.A.; Proost, J.H.; Cocolo, A.; Passamonti, S.; Groothuis, G.M. Bilitranslocase is involved in the uptake of bromosulfophthalein in rat and human liver. *Drug Metab. Lett.* **2012**, *6*, 165–173.
5. Passamonti, S.; Terdoslavich, M.; Margon, A.; Cocolo, A.; Medic, N.; Micali, F.; Decorti, G.; Franko, M. Uptake of bilirubin into HepG2 cells assayed by thermal lens spectroscopy. *FEBS J.* **2005**, *272*, 5522–5535.
6. Montanic, S.; Terdoslavich, M.; Rajcevic, U.; De Leo, L.; Curin Serbec, V.; Passamonti, S. Development and characterization of a novel mAb against bilitranslocase-a new biomarker of renal carcinoma. *Radiol. Oncol.* **2013**, *47*, 128–137.
7. Župerl, Š.; Fornasaro, S.; Novič, M.; Passamonti, S. Experimental determination and prediction of bilitranslocase transport activity. *Anal. Chim. Acta* **2011**, *705*, 322–333.
8. Battiston, L.; Passamonti, S.; Macagno, A.; Sottocasa, G.L. The bilirubin-binding motif of bilitranslocase and its relation to conserved motifs in ancient biliproteins. *Biochem. Biophys. Res. Commun.* **1998**, *247*, 687–692.
9. Passamonti, S.; Battiston, L.; Sottocasa, G.L. Bilitranslocase can exist in two metastable forms with different affinities for the substrates. *FEBS J.* **1998**, *253*, 84–90.
10. Karawajczyk, A.; Drgan, V.; Medic, N.; Oboh, G.; Passamonti, S.; Novič, M. Properties of flavonoids influencing the binding to bilitranslocase investigated by neural network modelling. *Biochem. Pharmacol.* **2007**, *73*, 308–320.
11. Passamonti, S.; Cocolo, A.; Braidot, E.; Petrussa, E.; Peresson, C.; Medic, N.; Macri, F.; Vianello, A. Characterization of electrogenic bromosulfophthalein transport in carnation petal microsomes and its inhibition by antibodies against bilitranslocase. *FEBS J.* **2005**, *272*, 3282–3296.
12. Maestro, A.; Terdoslavich, M.; Vanzo, A.; Kuku, A.; Tramer, F.; Nicolin, V.; Micali, F.; Decorti, G.; Passamonti, S. Expression of bilitranslocase in the vascular endothelium and its function as a flavonoid transporter. *Cardiovasc. Res.* **2009**, *85*, 175–183.

13. Ziberna, L.; Tramer, F.; Moze, S.; Vrhovsek, U.; Mattivi, F.; Passamonti, S. Transport and bioactivity of cyanidin 3-glucoside into the vascular endothelium. *Free Radic. Biol. Med.* **2012**, *52*, 1750–1759.
14. Roy Choudhury, A.; Novič, M. Data-driven model for the prediction of protein transmembrane regions. *SAR QSAR Environ. Res.* **2009**, *20*, 741–754.
15. Perdih, A.; Choudhury, A.R.; Župerl, Š.; Sikorska, E.; Zhukov, I.; Solmajer, T.; Novič, M. Structural analysis of a peptide fragment of transmembrane transporter protein bilitranslocase. *PLoS ONE* **2012**, *7*, e38967.
16. Choudhury, A.R.; Perdih, A.; Župerl, Š.; Sikorska, E.; Solmajer, T.; Jurga, S.; Zhukov, I.; Novič, M. Structural elucidation of transmembrane transporter protein Bilitranslocase: Conformational analysis of the second transmembrane region TM2 by molecular dynamics and NMR spectroscopy. *Biochim. Biophys. Acta (BBA) Biomembr.* **2013**, *1828*, 2609–2619.
17. Choudhury, A.R.; Sikorska, E.; van den Boom, J.; Bayer, P.; Popenda, Ł.; Szutkowski, K.; Jurga, S.; Bonomi, M.; Sali, A.; Zhukov, I.; et al. Structural model of the bilitranslocase transmembrane domain supported by NMR and FRET data. *PLoS ONE* **2015**, *10*, e0135455.
18. Pieper, U.; Schlessinger, A.; Kloppmann, E.; Chang, G.A.; Chou, J.J.; Dumont, M.E.; Fox, B.G.; Fromme, P.; Hendrickson, W.A.; Malkowski, M.G.; et al. Coordinating the impact of structural genomics on the human α-helical transmembrane proteome. *Nat. Struct. Mol. Biol.* **2013**, *20*, 135–138.
19. Vashisth, H.; Storaska, A.J.; Neubig, R.R.; Brooks, C.L., III. Conformational dynamics of a regulator of G-protein signaling protein reveals a mechanism of allosteric inhibition by a small molecule. *ACS Chem. Biol.* **2013**, *8*, 2778–2784.
20. Schmidpeter, P.A.; Schmid, F.X. Molecular determinants of a regulatory prolyl isomerization in the signal adapter protein c-CrkII. *ACS Chem. Biol.* **2014**, *9*, 1145–1152.
21. Nugent, T.; Jones, D.T. Membrane protein structural bioinformatics. *J. Struct. Biol.* **2012**, *179*, 327–337.
22. Choudhury, A.R.; Novič, M. PredβTM: A novel β-transmembrane region prediction algorithm. *PLoS ONE* **2015**, *10*, e0145564.
23. Nieh, M.P.; Raghunathan, V.A.; Kline, S.R.; Harroun, T.A.; Huang, C.Y.; Pencer, J.; Katsaras, J. Spontaneously formed unilamellar vesicles with path-dependent size distribution. *Langmuir* **2005**, *21*, 6656–6661.
24. Kallick, D.A.; Tessmer, M.R.; Watts, C.R.; Li, C.Y. The use of dodecylphosphocholine micelles in solution NMR. *J. Magn. Reson. Ser. B* **1995**, *109*, 60–65.
25. Chipot, C.; Dehez, F.; Schnell, J.R.; Zitzmann, N.; Pebay-Peyroula, E.; Catoire, L.J.; Miroux, B.; Kunji, E.R.; Veglia, G.; Cross, T.A.; et al. Perturbations of native membrane protein structure in alkyl phosphocholine detergents: A critical assessment of NMR and biophysical studies. *Chem. Rev.* **2018**, *118*, 3559–3607.
26. Zhou, H.X.; Cross, T.A. Modeling the membrane environment has implications for membrane protein structure and function: Influenza A M2 protein. *Protein Sci.* **2013**, *22*, 381–394.
27. Hofmann, K. TMbase-A database of membrane spanning proteins segments. *Biol. Chem. Hoppe-Seyler* **1993**, *374*, 166.
28. Claros, M.G.; von Heijne, G. TopPred II: An improved software for membrane protein structure predictions. *Bioinformatics* **1994**, *10*, 685–686.
29. Pasquier, C.; Promponas, V.; Palaios, G.; Hamodrakas, J.; Hamodrakas, S. A novel method for predicting transmembrane segments in proteins based on a statistical analysis of the SwissProt database: The PRED-TMR algorithm. *Protein Eng.* **1999**, *12*, 381–385.
30. Shen, H.; Chou, J.J. MemBrain: Improving the accuracy of predicting transmembrane helices. *PLoS ONE* **2008**, *3*, e2399.
31. Reynolds, S.M.; Käll, L.; Riffle, M.E.; Bilmes, J.A.; Noble, W.S. Transmembrane topology and signal peptide prediction using dynamic bayesian networks. *PLoS Comput. Biol.* **2008**, *4*, e1000213.
32. Tusnady, G.E.; Simon, I. The HMMTOP transmembrane topology prediction server. *Bioinformatics* **2001**, *17*, 849–850.
33. Bernsel, A.; Viklund, H.; Falk, J.; Lindahl, E.; von Heijne, G.; Elofsson, A. Prediction of membrane-protein topology from first principles. *Proc. Natl. Acad. Sci. USA* **2008**, *105*, 7177–7181.
34. Bernsel, A.; Viklund, H.; Hennerdal, A.; Elofsson, A. TOPCONS: Consensus prediction of membrane protein topology. *Nucleic Acids Res.* **2009**, *37*, W465–W468.
35. Hirokawa, T.; Boon-Chieng, S.; Mitaku, S. SOSUI: Classification and secondary structure prediction system for membrane proteins. *Bioinformatics* **1998**, *14*, 378–379.
36. Humphrey, W.; Dalke, A.; Schulten, K. VMD: Visual molecular dynamics. *J. Mol. Graph.* **1996**, *14*, 33–38.

37. Page, R.C.; Kim, S.; Cross, T.A. Transmembrane helix uniformity examined by spectral mapping of torsion angles. *Structure* **2008**, *16*, 787–797.
38. Luo, P.; Baldwin, R.L. Mechanism of helix induction by trifluoroethanol: A framework for extrapolating the helix-forming properties of peptides from trifluoroethanol/water mixtures back to water. *Biochemistry* **1997**, *36*, 8413–8421.
39. Baldwin, R.L. Temperature dependence of the hydrophobic interaction in protein folding. *Proc. Natl. Acad. Sci. USA* **1986**, *83*, 8069–8072.
40. Mu, Y.; Gao, Y.Q. Effects of hydrophobic and dipole-dipole interactions on the conformational transitions of a model polypeptide. *J. Chem. Phys.* **2007**, *127*, 09B604.
41. Sreerama, N.; Woody, R.W. On the analysis of membrane protein circular dichroism spectra. *Protein Sci.* **2004**, *13*, 100–112.
42. Miles, A.J.; Wallace, B.A. Circular dichroism spectroscopy of membrane proteins. *Chem. Soc. Rev.* **2016**, *45*, 4859–4872.
43. Wüthrich, K. NMR with Proteins and Nucleic Acids. *Europhys. News* **1986**, *17*, 11–13.
44. Güntert, P.; Buchner, L. Combined automated NOE assignment and structure calculation with CYANA. *J. Biomol. NMR* **2015**, *62*, 453–471.
45. Shen, Y.; Bax, A. Protein structural information derived from NMR chemical shift with the neural network program TALOS-N. In *Artificial Neural Networks*; Springer: New York, NY, USA, 2015; pp. 17–32.
46. Kholodenko, A.L.; Douglas, J.F. Generalized Stokes-Einstein equation for spherical particle suspensions. *Phys. Rev. E* **1995**, *51*, 1081.
47. Cantor, C.R.; Schimmel, P.R. *Biophysical Chemistry, Part 2: Techniques for the Study of Biological Structure and Function, Part 2*; Freeman: San Francisco, CA, USA, 1980.
48. Chou, J.J.; Baber, J.L.; Bax, A. Characterization of phospholipid mixed micelles by translational diffusion. *J. Biomol. NMR* **2004**, *29*, 299–308.
49. Beswick, V.; Guerois, R.; Cordier-Ochsenbein, F.; Coic, Y.M.; Huynh-Dinh, T.; Tostain, J.; Noel, J.P.; Sanson, A.; Neumann, J.M. Dodecylphosphocholine micelles as a membrane-like environment: New results from NMR relaxation and paramagnetic relaxation enhancement analysis. *Eur. Biophys. J.* **1998**, *28*, 48–58.
50. Jafari, M.; Mehrnejad, F.; Doustdar, F. Insight into the interactions, residue snorkeling, and membrane disordering potency of a single antimicrobial peptide into different lipid bilayers. *PLoS ONE* **2017**, *12*, e0187216.
51. Szutkowski, K.; Stilbs, P.; Jurga, S. Proton chemical exchange in aqueous solutions of dodecylammonium chloride: Effects of micellar aggregation. *J. Phys. Chem. C* **2007**, *111*, 15613–15619.
52. Carver, J.; Richards, R. A general two-site solution for the chemical exchange produced dependence of T_2 upon the Carr-Purcell pulse separation. *J. Magn. Reson.* **1972**, *6*, 89–105.
53. Palmer, A.G., III; Kroenke, C.D.; Loria, J.P. Nuclear magnetic resonance methods for quantifying microsecond-to-millisecond motions in biological macromolecules. *Methods Enzymol.* **2001**, *339*, 204–238.
54. Langham, A.A.; Waring, A.J.; Kaznessis, Y. Comparison of interactions between beta-hairpin decapeptides and SDS/DPC micelles from experimental and simulation data. *BMC Biochem.* **2007**, *8*, 11.
55. Venko, K.; Choudhury, A.R.; Novič, M. Computational approaches for revealing the structure of membrane transporters: Case study on bilitranslocase. *Comput. Struct. Biotechnol. J.* **2017**, *15*, 232–242.
56. Nogales, D.; Lightner, D.A. On the structure of bilirubin in solution. *J. Biol. Chem.* **1995**, *270*, 73–77.
57. Xie, M.; Holmes, D.L.; Lightner, D.A. Bilirubin conformation and intramolecular steric buttressing. C(10)-*gem*-dimethyl effect. *Tetrahedron* **1993**, *49*, 9235–9250.
58. Krogh, A.; Larsson, B.; von Heijne, G.; Sonnhammer, E.L. Predicting transmembrane protein topology with a hidden Markov model: application to complete genomes. *J. Mol. Biol.* **2001**, *305*, 567–580.
59. Käll, L.; Krogh, A.; Sonnhammer, E.L. A combined transmembrane topology and signal peptide prediction method. *J. Mol. Biol.* **2004**, *338*, 1027–1036.
60. Yuan, Z.; Mattick, J.S.; Teasdale, R.D. SVMtm: Support vector machines to predict transmembrane segments. *J. Comput. Chem.* **2004**, *25*, 632–636.
61. Cserzo, M.; Eisenhaber, F.; Eisenhaber, B.; Simon, I. TM or not TM: Transmembrane protein prediction with low false positive rate using DAS-TMfilter. *Bioinformatics* **2004**, *20*, 136–137.
62. Jones, D.T. Improving the accuracy of transmembrane protein topology prediction using evolutionary information. *Bioinformatics* **2007**, *23*, 538–544.

63. Viklund, H.; Elofsson, A. A method that improves topology prediction for transmembrane proteins by using two-track ANN-based preference scores and an improved topological grammar. *Bioinformatics* **2008**, *24*, 1662–1668.
64. Brooks, B.R.; Brooks, C.L., III; MacKerell, A.D., Jr.; Nilsson, L.; Petrella, R.J.; Roux, B.; Won, Y.; Archontis, G.; Bartels, C.; Boresch, S.; et al. CHARMM: The biomolecular simulation program. *J. Comput. Chem.* **2009**, *30*, 1545–1614.
65. MacKerell, A., Jr.; Bashford, D.; Bellott, M.; Dunbrack, R., Jr.; Evanseck, J.; Field, M.; Fischer, S.; Gao, J.; Guo, H.; Ha, S.; et al. All-atom empirical potential for molecular modeling and dynamics studies of proteins. *J. Phys. Chem. B* **1998**, *102*, 3586.
66. Klauda, J.B.; Venable, R.M.; Freites, J.A.; O'Connor, J.W.; Tobias, D.J.; Mondragon-Ramirez, C.; Vorobyov, I.; MacKerell, A.D., Jr.; Pastor, R.W. Update of the CHARMM all-atom additive force field for lipids: validation on six lipid types. *J. Phys. Chem. B* **2010**, *114*, 7830–7843.
67. Frishman, D.; Argos, P. Knowledge-based protein secondary structure assignment. *Proteins Struct. Funct. Bioinform.* **1995**, *23*, 566–579.
68. Racine, J. gnuplot 4.0: A portable interactive plotting utility. *J. Appl. Econom.* **2006**, *21*, 133–141.
69. Turro, N.J.; Yekta, A. Luminescent probes for detergent solutions. A simple procedure for determination of the mean aggregation number of micelles. *J. Am. Chem. Soc.* **1978**, *100*, 5951–5952.
70. Hsu, S.T.D.; Breukink, E.; Bierbaum, G.; Sahl, H.G.; de Kruijff, B.; Kaptein, R.; van Nuland, N.A.; Bonvin, A.M. NMR study of Mersacidin and lipid II interaction in dodecylphosphocholine micelles. *J. Biol. Chem.* **2003**, *278*, 13110–13117.
71. Hwang, T.L.; Shaka, A. Water suppression that works. Excitation sculpting using arbitrary wave-forms and pulsed-field gradients. *J. Magn. Reson. Ser. A* **1995**, *112*, 275–279.
72. Wishart, D.S.; Bigam, C.G.; Yao, J.; Abildgaard, F.; Dyson, H.J.; Oldfield, E.; Markley, J.L.; Sykes, B.D. ^{1}H, ^{13}C and ^{15}N chemical shift referencing in biomolecular NMR. *J. Biomol. NMR* **1995**, *6*, 135–140.
73. Delaglio, F.; Grzesiek, S.; Vuister, G.W.; Zhu, G.; Pfeifer, J.; Bax, A. NMRPipe: A multidimensional spectral processing system based on UNIX pipes. *J. Biomol. NMR* **1995**, *6*, 277–293.
74. Lee, W.; Tonelli, M.; Markley, J.L. NMRFAM-SPARKY: Enhanced software for biomolecular NMR spectroscopy. *Bioinformatics* **2014**, *31*, 1325–1327.
75. Shen, Y.; Bax, A. Prediction of Xaa-Pro peptide bond conformation from sequence and chemical shifts. *J. Biomol. NMR* **2010**, *46*, 199–204.
76. Sharma, D.; Rajarathnam, K. ^{13}C NMR chemical shifts can predict disulfide bond formation. *J. Biomol. NMR* **2000**, *18*, 165–171.
77. Case, D.A.; Darden, T.; Cheatham, T.E., III; Simmerling, C.; Wang, J.; Duke, R.E.; Luo, R.; Merz, K.M.; Pearlman, D.A.; Crowley, M.; et al. *AMBER 9*; University of California: San Francisco, CA, USA, 2006.
78. Case, D.A.; Babin, V.; Berryman, J.; Betz, R.M.; Cai, Q.; Cerutti, D.S.; Cheatham, T.E., III; Darden, T.A.; Duke, R.E.; Gohlke, H.; et al. *AMBER 14, 2014*; University of California: San Francisco, CA, USA, 2014.
79. Koradi, R.; Billeter, M.; Wüthrich, K. MOLMOL: A program for display and analysis of macromolecular structures. *J. Mol. Graph.* **1996**, *14*, 51–55.
80. Pettersen, E.F.; Goddard, T.D.; Huang, C.C.; Couch, G.S.; Greenblatt, D.M.; Meng, E.C.; Ferrin, T.E. UCSF Chimera—A visualization system for exploratory research and analysis. *J. Comput. Chem.* **2004**, *25*, 1605–1612.
81. Nilsson, M.; Gil, A.M.; Delgadillo, I.; Morris, G.A. Improving pulse sequences for 3D diffusion-ordered NMR spectroscopy: 2DJ-IDOSY. *Anal. Chem.* **2004**, *76*, 5418–5422.
82. Wu, D.; Chen, A.; Johnson, C.S. An improved diffusion-ordered spectroscopy experiment incorporating bipolar-gradient pulses. *J. Magn. Reson. Ser. A* **1995**, *115*, 260–264.
83. Scotti, A.; Liu, W.; Hyatt, J.; Herman, E.; Choi, H.; Kim, J.; Lyon, L.; Gasser, U.; Fernandez-Nieves, A. The CONTIN algorithm and its application to determine the size distribution of microgel suspensions. *J. Chem. Phys.* **2015**, *142*, 234905.
84. Stejskal, E.O.; Tanner, J.E. Spin diffusion measurements: Spin echoes in the presence of a time-dependent field gradient. *J. Chem. Phys.* **1965**, *42*, 288–292.
85. Farrow, N.A.; Muhandiram, R.; Singer, A.U.; Pascal, S.M.; Kay, C.M.; Gish, G.; Shoelson, S.E.; Pawson, T.; Forman-Kay, J.D.; Kay, L.E. Backbone dynamics of a free and a phosphopeptide-complexed Src homology 2 domain studied by ^{15}N NMR relaxation. *Biochemistry* **1994**, *33*, 5984–6003.

86. Kay, L.E.; Nicholson, L.K.; Delaglio, F.; Bax, A.; Torchia, D.A. Pulse sequences for removal of the effects of cross correlation between dipolar and chemical-shift anisotropy relaxation mechanisms on the measurement of heteronuclear T_1 and T_2 values in proteins. *J. Magn. Reson.* **1992**, *97*, 359–375.
87. Li, C.; Gao, P.; Qin, H.; Chase, R.; Gor'kov, P.L.; Brey, W.W.; Cross, T.A. Uniformly aligned full-length membrane proteins in liquid crystalline bilayers for structural characterization. *J. Am. Chem. Soc.* **2007**, *129*, 5304–5305.
88. Sreerama, N.; Woody, R.W. Estimation of protein secondary structure from circular dichroism spectra: Comparison of CONTIN, SELCON, and CDSSTR methods with an expanded reference set. *Anal. Biochem.* **2000**, *287*, 252–260.
89. Jo, S.; Kim, T.; Iyer, V.G.; Im, W. CHARMM-GUI: A web-based graphical user interface for CHARMM. *J. Comput. Chem.* **2008**, *29*, 1859–1865.
90. Vriend, G. WHAT IF: A molecular modeling and drug design program. *J. Mol. Graph.* **1990**, *8*, 52–56.

© 2019 by the authors. Licensee MDPI, Basel, Switzerland. This article is an open access article distributed under the terms and conditions of the Creative Commons Attribution (CC BY) license (http://creativecommons.org/licenses/by/4.0/).
