# Supplementary figures and images for: Structural Analysis and Dynamic Processes of the Transmembrane Segment Inside Different Micellar Environments—Implications for the TM4 Fragment of the Bilitranslocase Protein
Source: Int J Mol Sci. 2019 Aug 26;20(17):4172. doi: 10.3390/ijms20174172 (PMC6747479; doi:10.3390/ijms20174172)

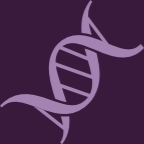

# International Journal of *Molecular Sciences*

Supplement: Supplementary file 1 [file ijms-20-04172-s001.zip › ijms-560636-S/Definitions/ijms-logo-eps-converted-to.pdf]

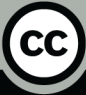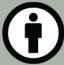

BY

Supplement: Supplementary file 1 [file ijms-20-04172-s001.zip › ijms-560636-S/Definitions/logo-ccby-eps-converted-to.pdf]

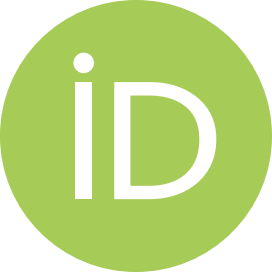

Supplement: Supplementary file 1 [file ijms-20-04172-s001.zip › ijms-560636-S/Definitions/logo-orcid-eps-converted-to.pdf]

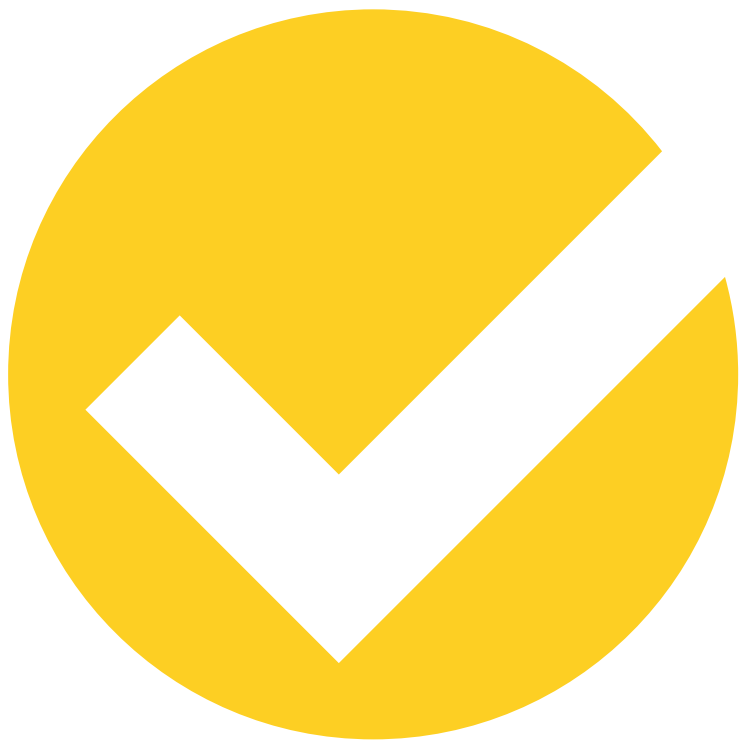

check for  
updates

Supplement: Supplementary file 1 [file ijms-20-04172-s001.zip › ijms-560636-S/Definitions/logo-updates.pdf]

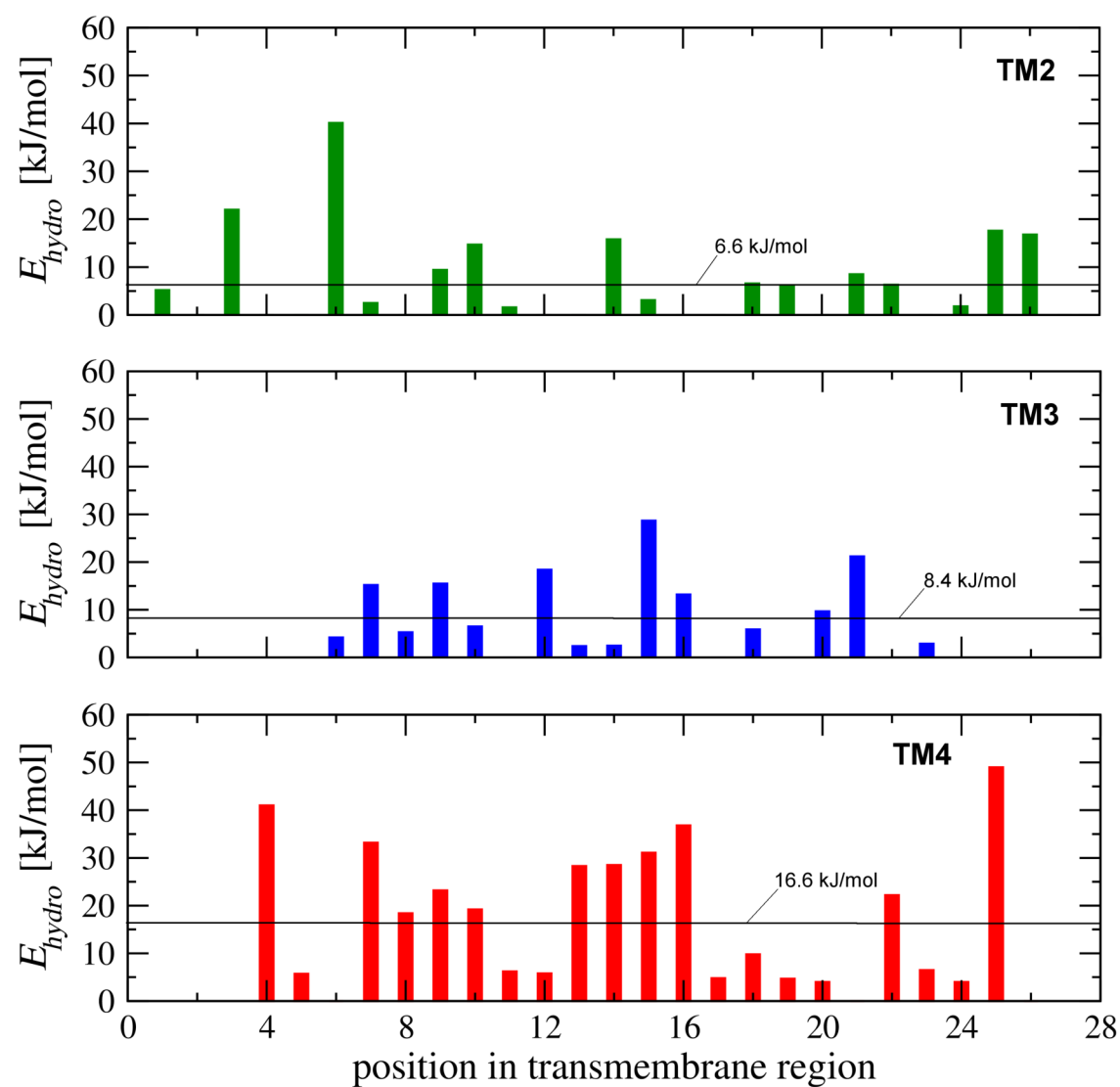

Supplement: Supplementary file 1 [file ijms-20-04172-s001.zip › ijms-560636-S/Supporting_Materials/tm2_tm3_tm4_sds_hydrophobic-eps-converted-to.pdf]

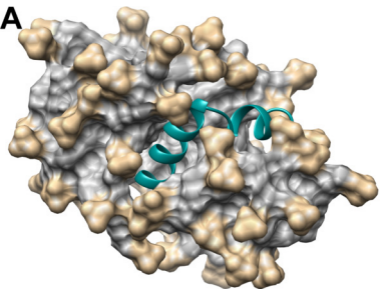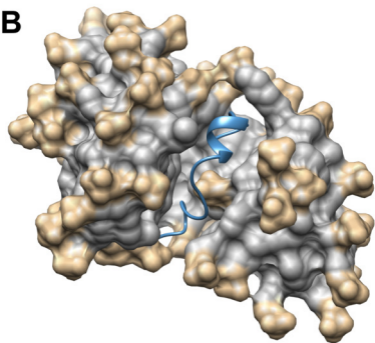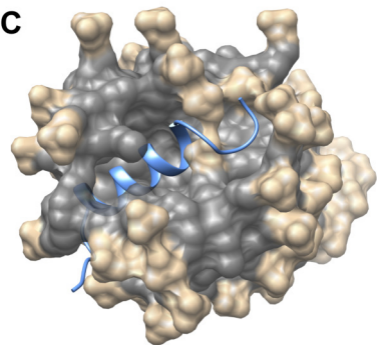

Supplement: Supplementary file 1 [file ijms-20-04172-s001.zip › ijms-560636-S/Supporting_Materials/tm2_tm3_tm4_sds_surface-eps-converted-to.pdf]

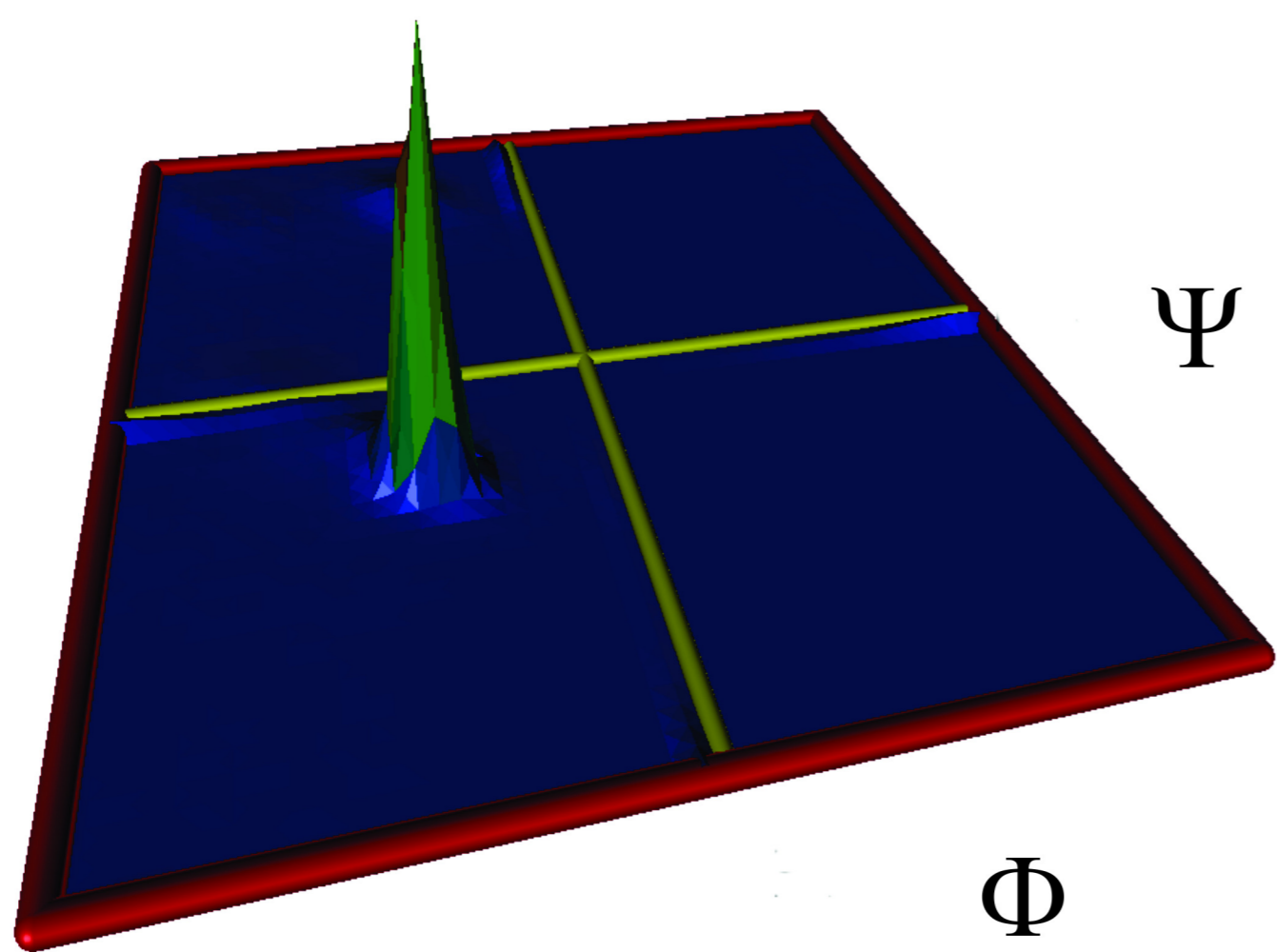

TM 4 HELIX

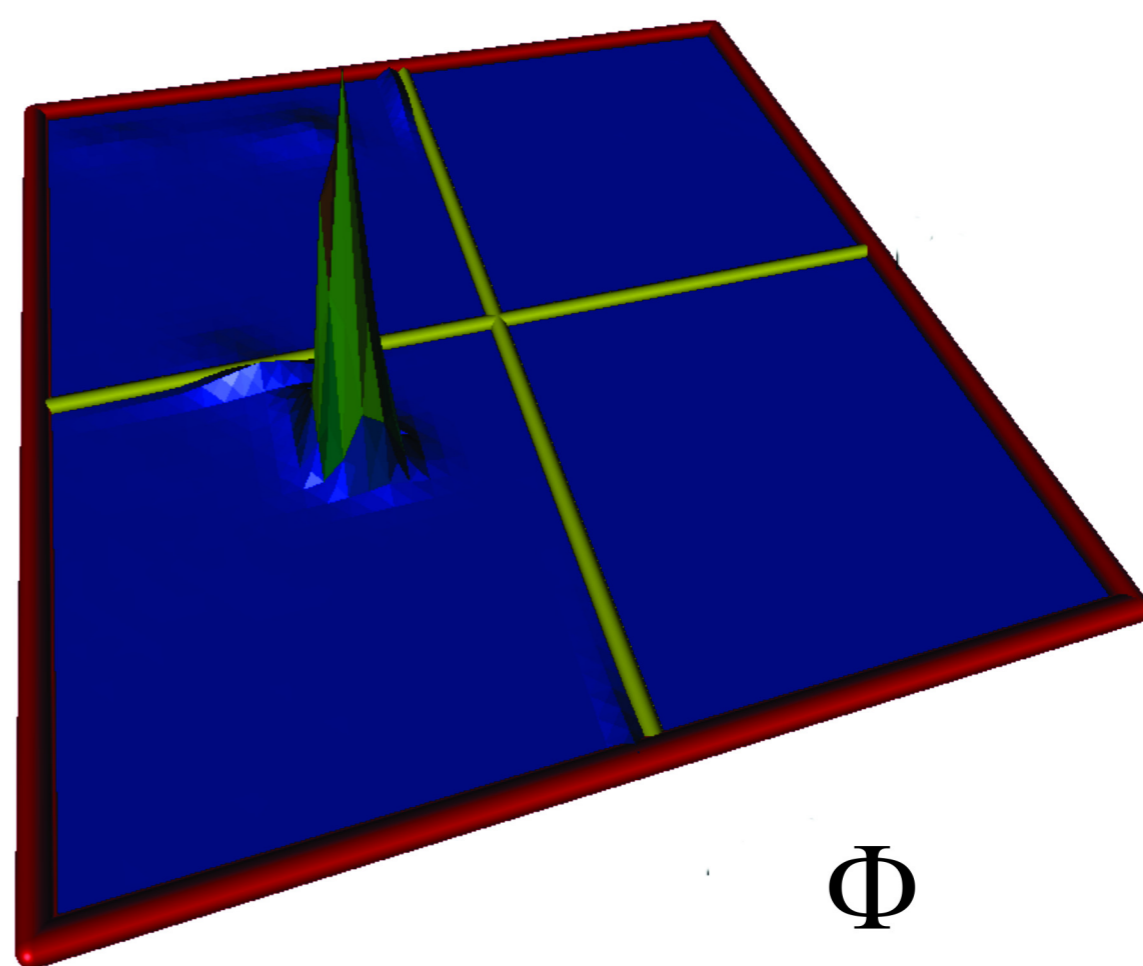

TM 4A HELIX

A

Ψ

Φ

Φ

B

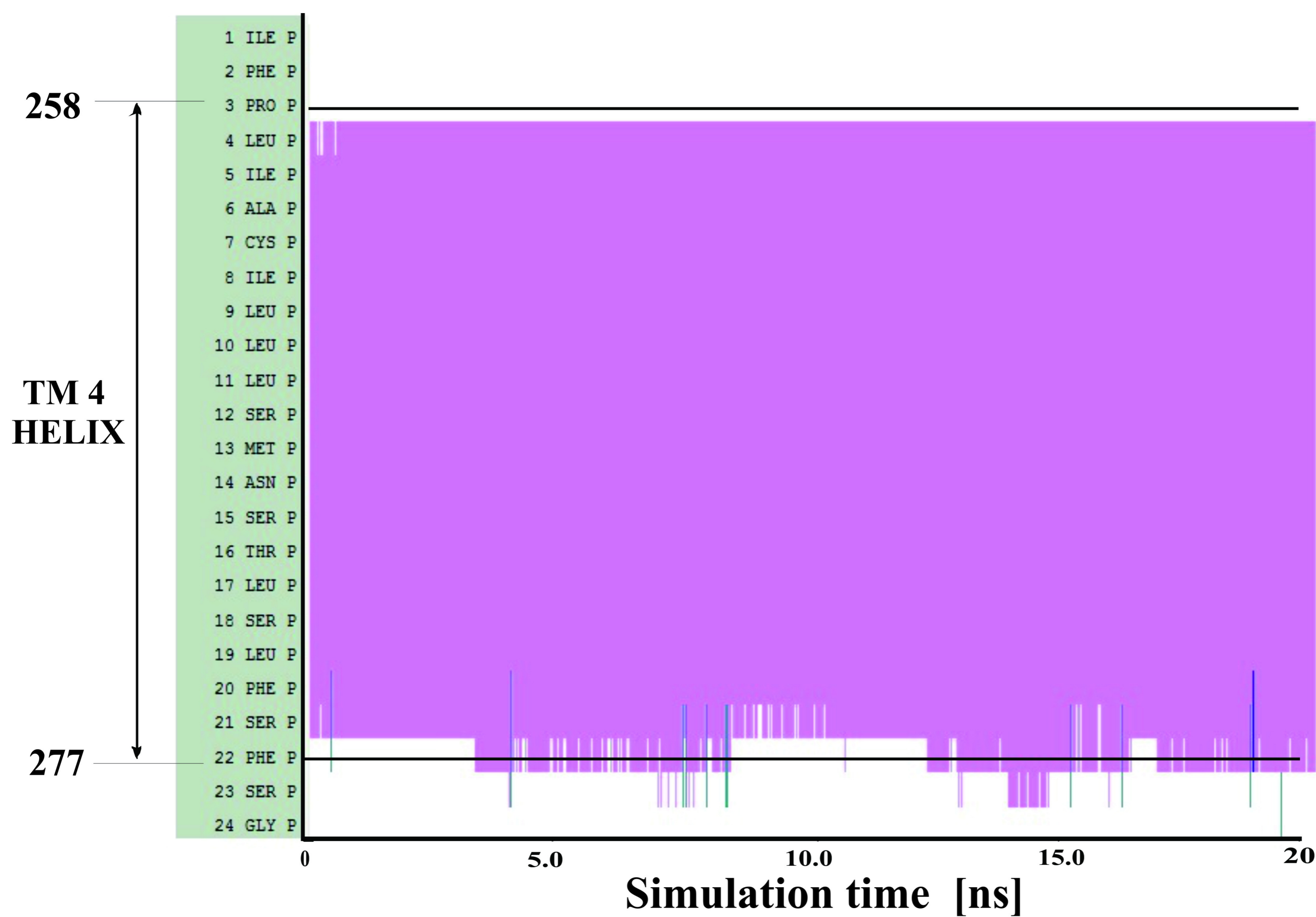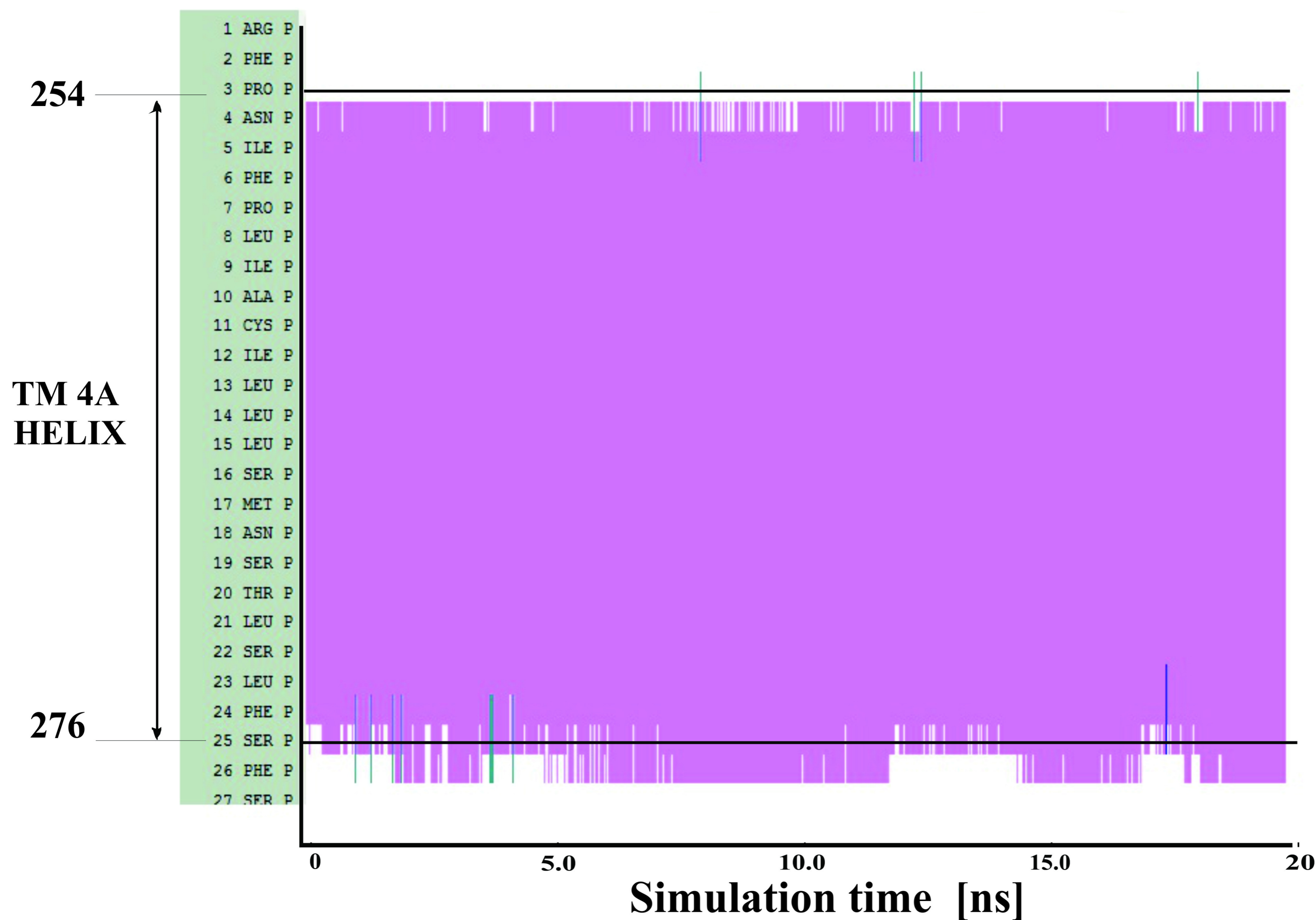

Supplement: Supplementary file 1 [file ijms-20-04172-s001.zip › ijms-560636-S/Supporting_Materials/tm4_3D_ramachandran_ap-eps-converted-to.pdf]

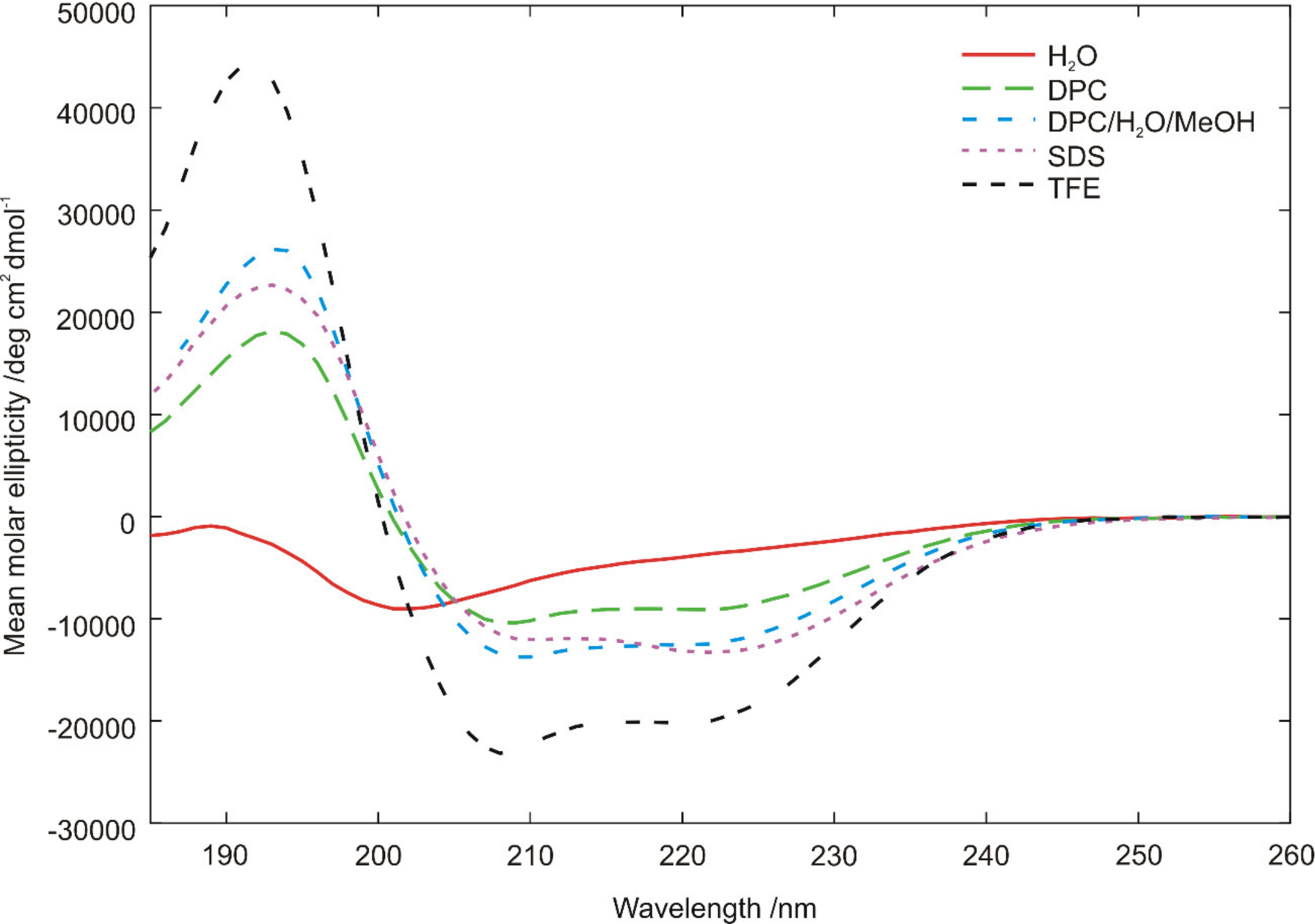

Supplement: Supplementary file 1 [file ijms-20-04172-s001.zip › ijms-560636-S/Supporting_Materials/tm4_CD_sds_dpc-eps-converted-to.pdf]

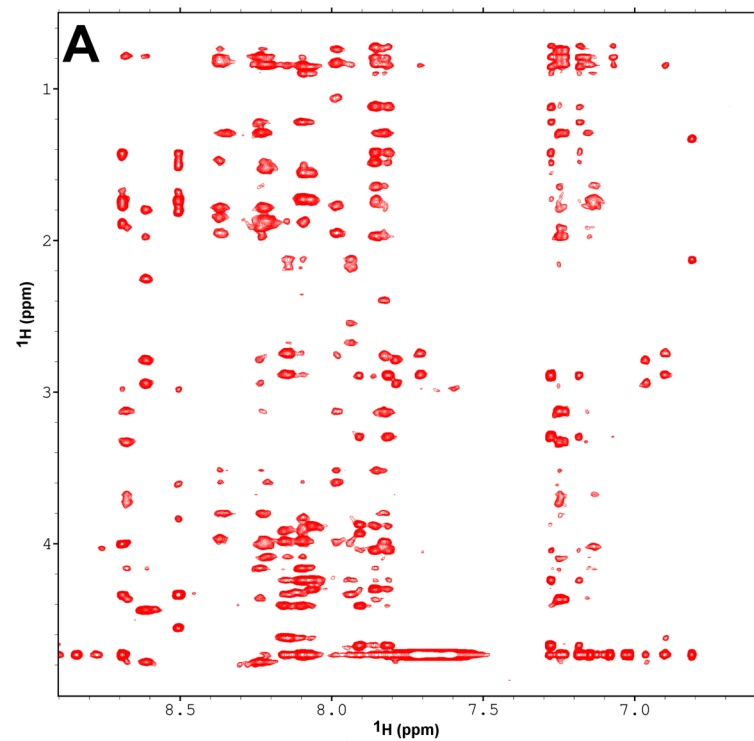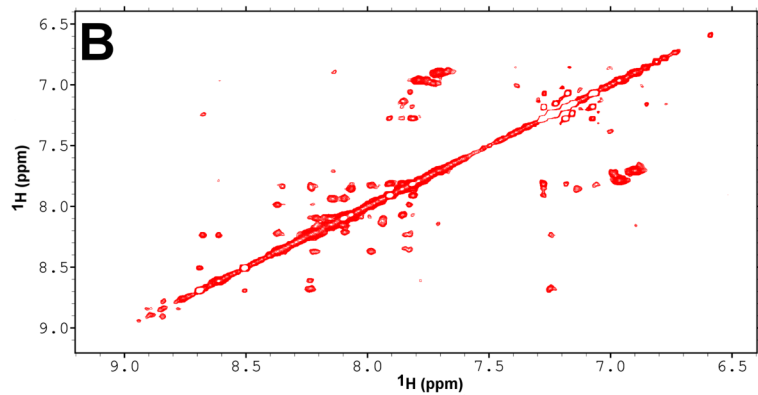

Supplement: Supplementary file 1 [file ijms-20-04172-s001.zip › ijms-560636-S/Supporting_Materials/tm4_dpc_noesy-eps-converted-to.pdf]

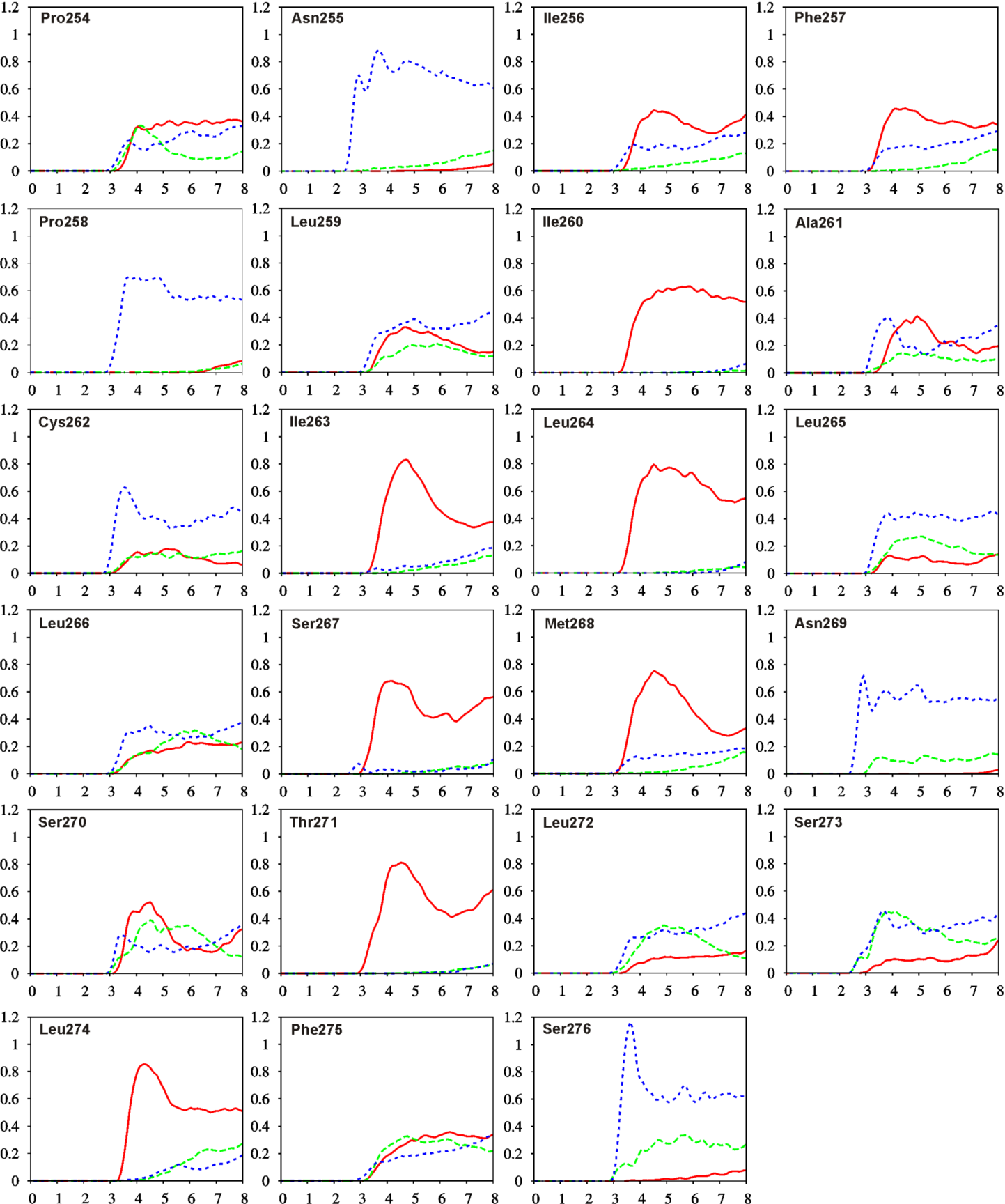

Supplement: Supplementary file 1 [file ijms-20-04172-s001.zip › ijms-560636-S/Supporting_Materials/tm4_dpc_rdf-eps-converted-to.pdf]

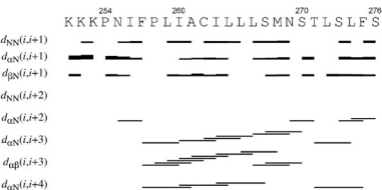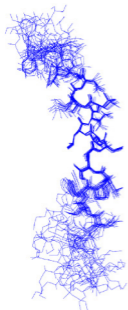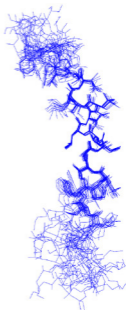

Supplement: Supplementary file 1 [file ijms-20-04172-s001.zip › ijms-560636-S/Supporting_Materials/tm4_dpc_stereo-eps-converted-to.pdf]

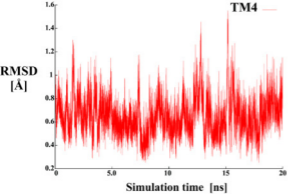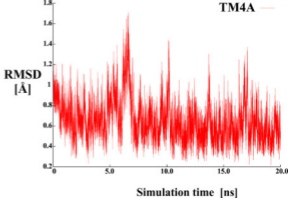

Supplement: Supplementary file 1 [file ijms-20-04172-s001.zip › ijms-560636-S/Supporting_Materials/tm4_rmsf_md-eps-converted-to.pdf]

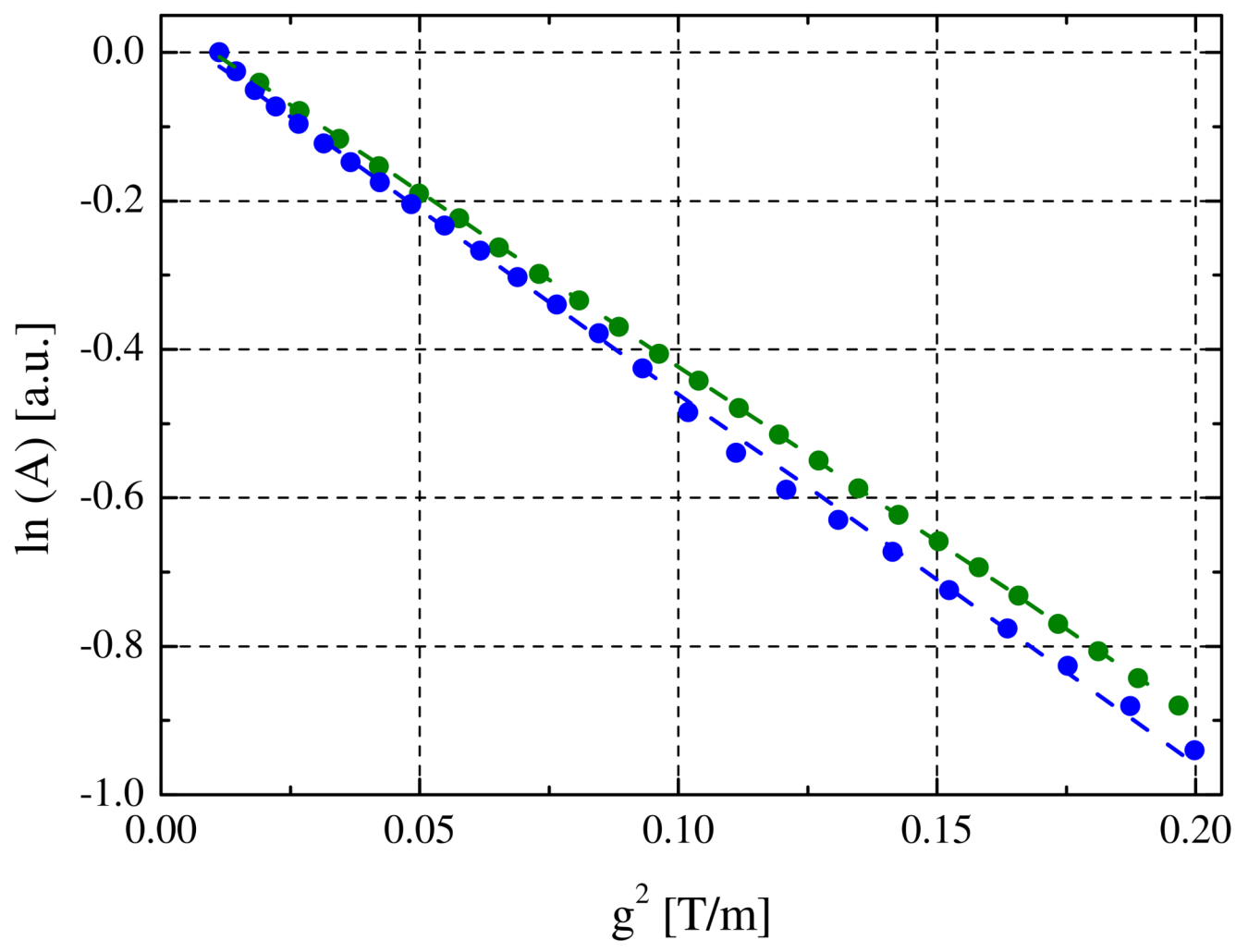

Supplement: Supplementary file 1 [file ijms-20-04172-s001.zip › ijms-560636-S/Supporting_Materials/tm4_sds_dpc_1H_diffusion-eps-converted-to.pdf]

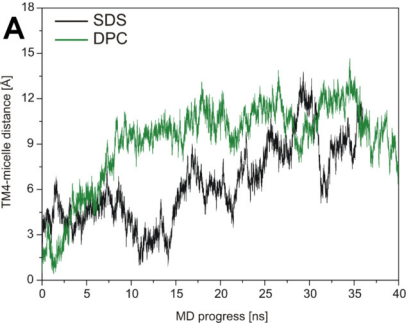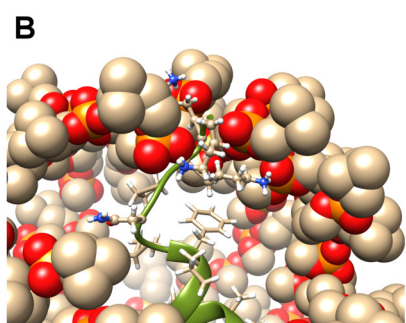

Supplement: Supplementary file 1 [file ijms-20-04172-s001.zip › ijms-560636-S/Supporting_Materials/tm4_sds_dpc_distance_dpc_snorkeling-eps-converted-to.pdf]

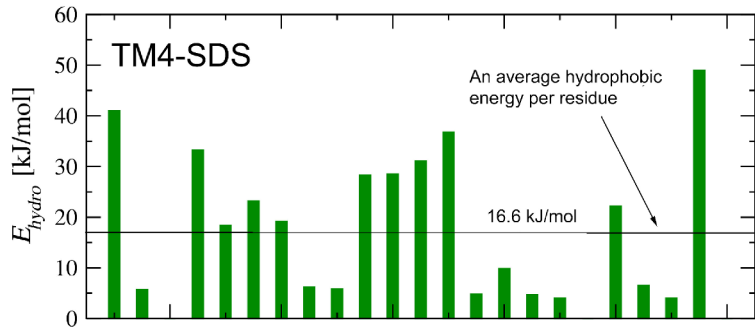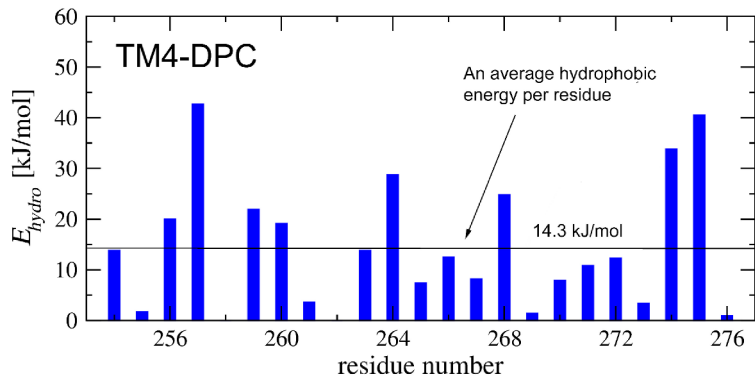

Supplement: Supplementary file 1 [file ijms-20-04172-s001.zip › ijms-560636-S/Supporting_Materials/tm4_sds_dpc_hydrophobic_contacts-eps-converted-to.pdf]

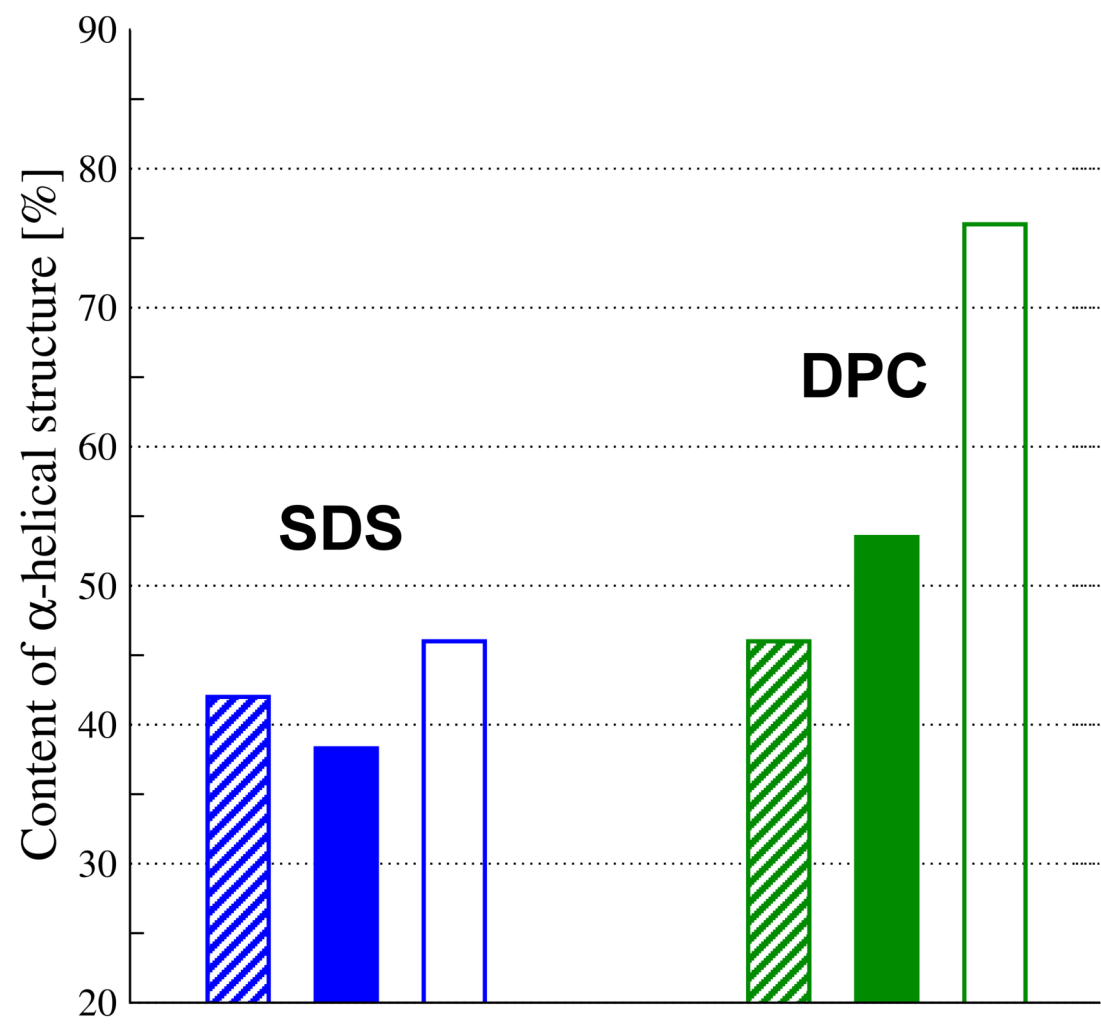

Supplement: Supplementary file 1 [file ijms-20-04172-s001.zip › ijms-560636-S/Supporting_Materials/tm4_sds_dpc_ribbon_content-eps-converted-to.pdf]

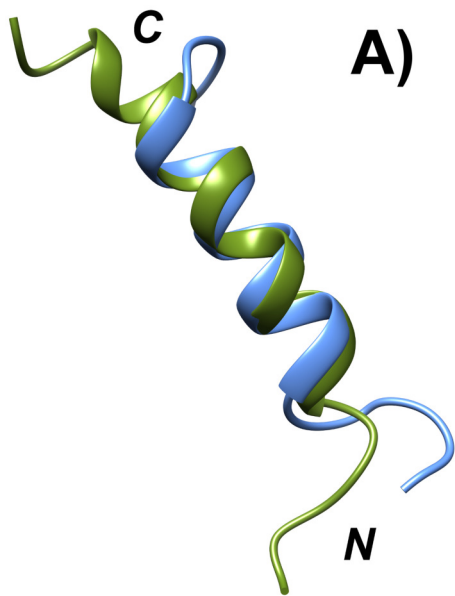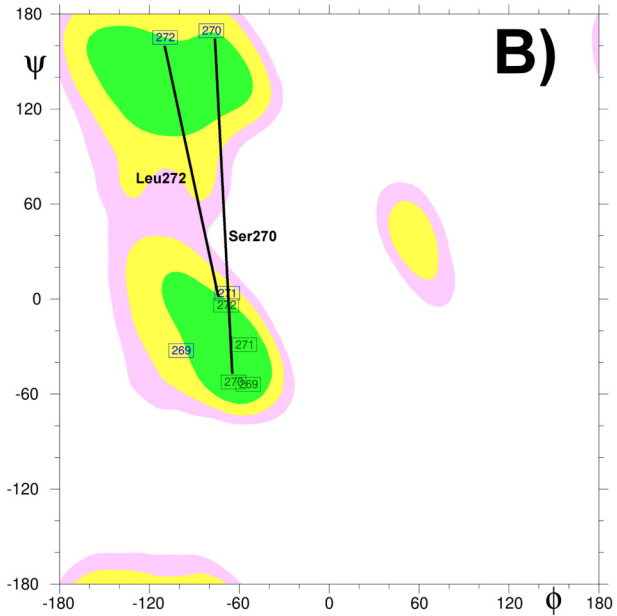

Supplement: Supplementary file 1 [file ijms-20-04172-s001.zip › ijms-560636-S/Supporting_Materials/tm4_sds_dpc_ribbon_overlay_rama-eps-converted-to.pdf]

# TM4 in SDS

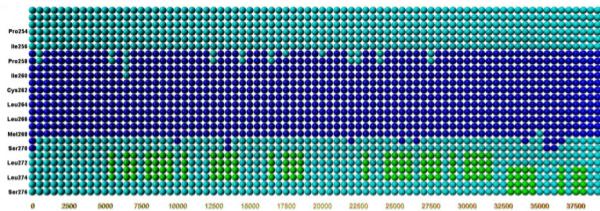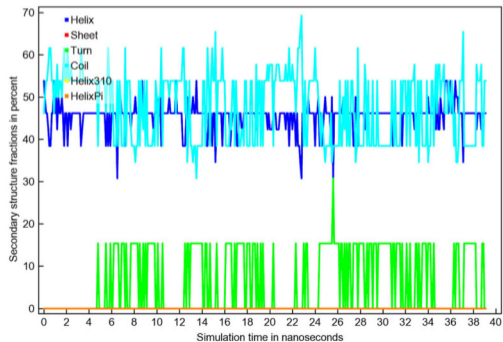

# TM4 in DPC

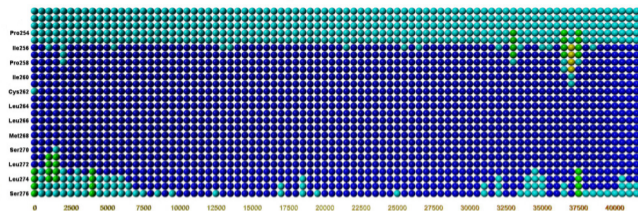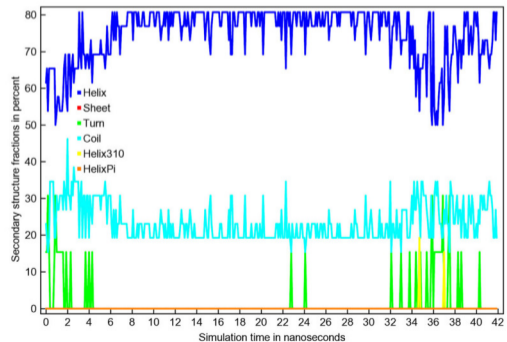

Supplement: Supplementary file 1 [file ijms-20-04172-s001.zip › ijms-560636-S/Supporting_Materials/tm4_sds_dpc_secondary_content-eps-converted-to.pdf]

**A**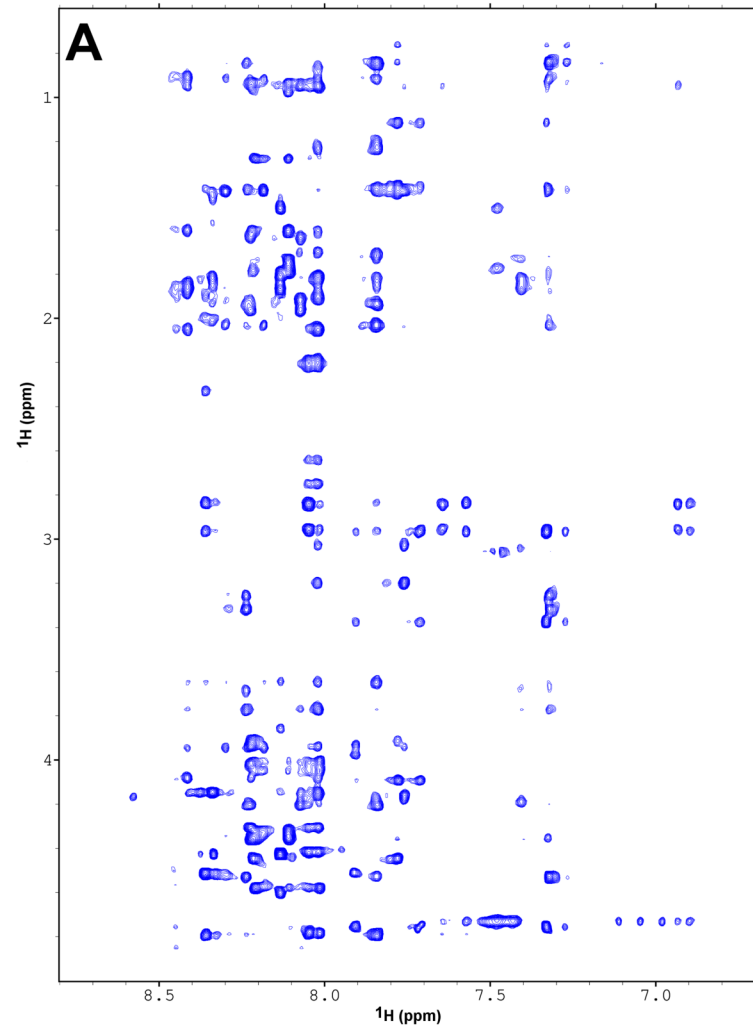**B**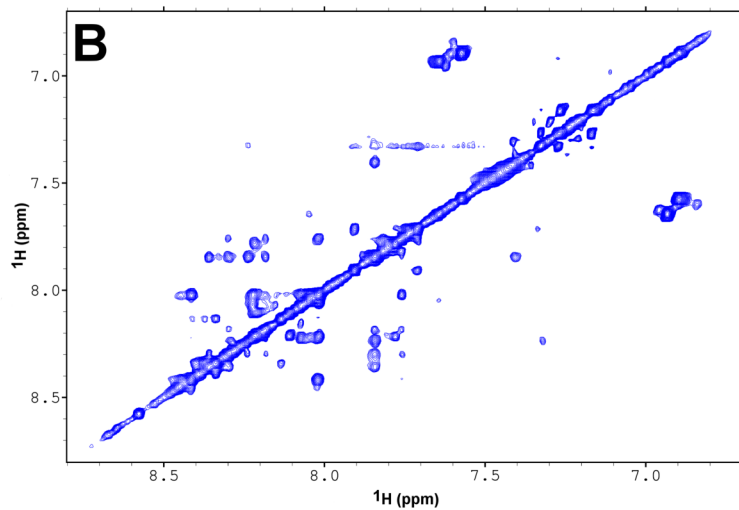

Supplement: Supplementary file 1 [file ijms-20-04172-s001.zip › ijms-560636-S/Supporting_Materials/tm4_sds_noesy-eps-converted-to.pdf]

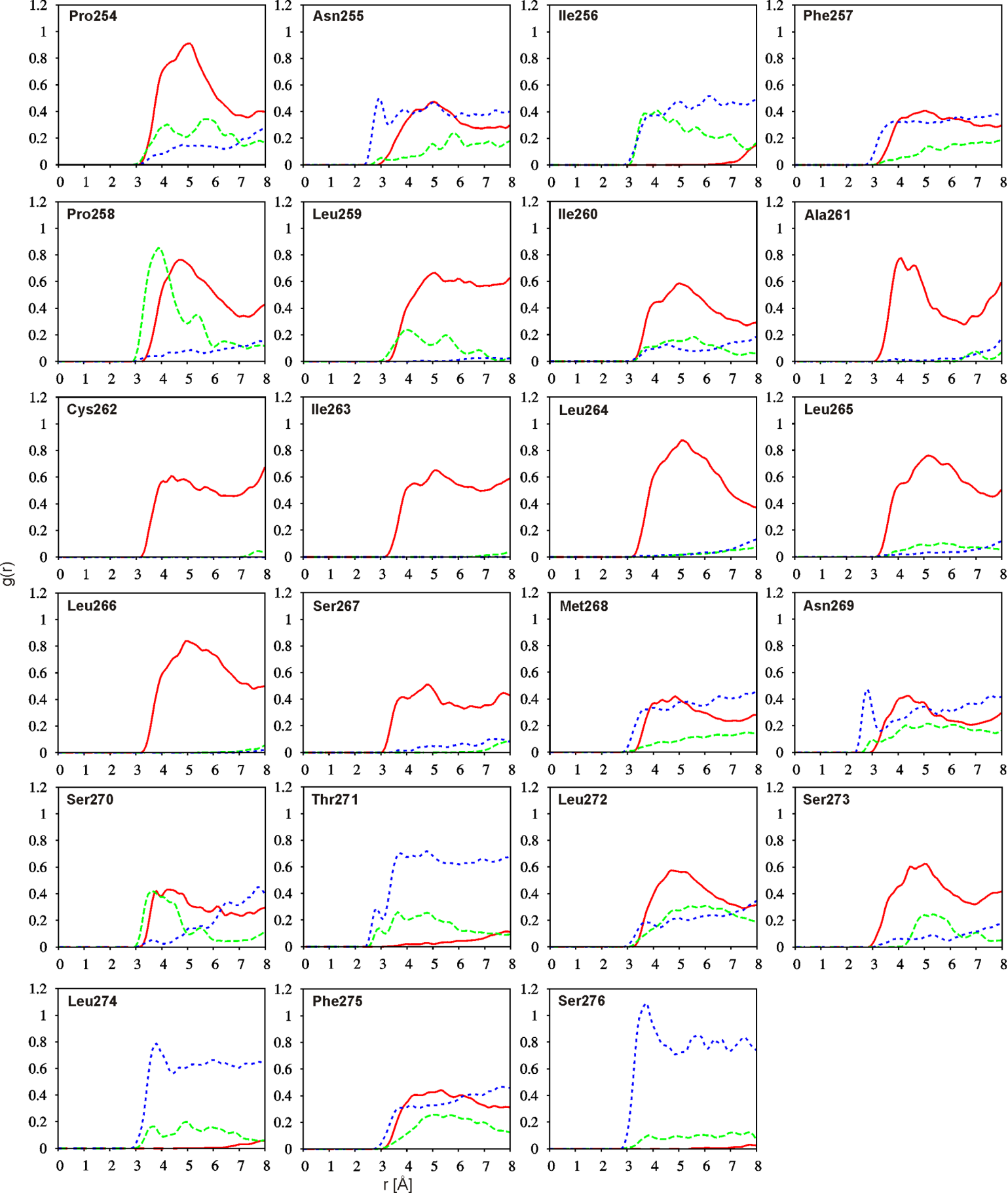

Supplement: Supplementary file 1 [file ijms-20-04172-s001.zip › ijms-560636-S/Supporting_Materials/tm4_sds_rdf-eps-converted-to.pdf]

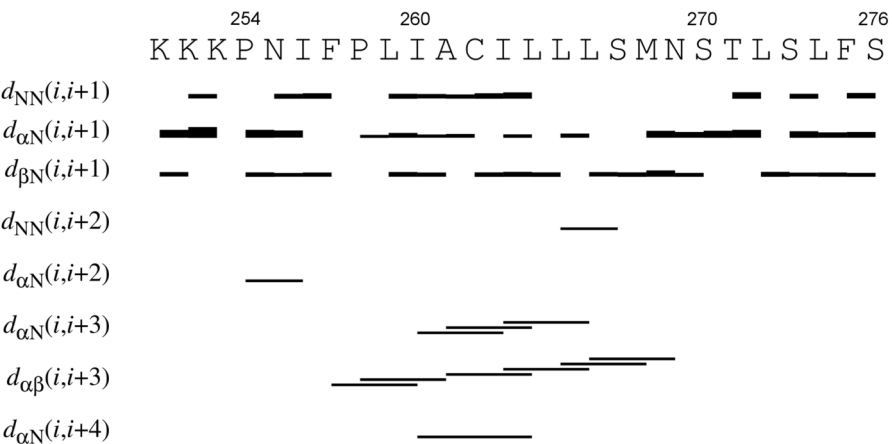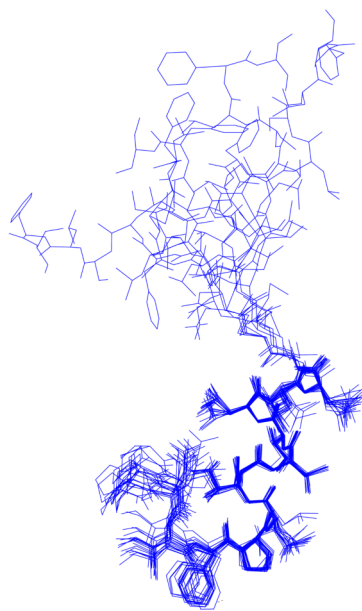

**Phe257**

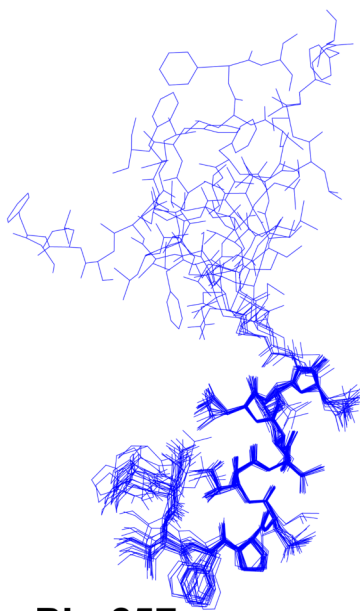

**Phe257**

Supplement: Supplementary file 1 [file ijms-20-04172-s001.zip › ijms-560636-S/Supporting_Materials/tm4_sds_stereo-eps-converted-to.pdf]
